# Classical complement and inflammasome activation converge in CD14^high^CD16^-^ monocytes in HIV associated TB-immune reconstitution inflammatory syndrome

**DOI:** 10.1371/journal.ppat.1009435

**Published:** 2021-03-31

**Authors:** Silvia Lucena Lage, Chun-Shu Wong, Eduardo Pinheiro Amaral, Daniel Sturdevant, Denise C. Hsu, Adam Rupert, Eleanor M. P. Wilson, S. Sonia Qasba, Nuha Sultana Naqvi, Elizabeth Laidlaw, Andrea Lisco, Maura Manion, Irini Sereti

**Affiliations:** 1 HIV Pathogenesis Section, Laboratory of Immunoregulation, National Institute of Allergy and Infectious Diseases, National Institutes of Health, Bethesda, MD, United States of America; 2 Immunobiology Section, Laboratory of Parasitic Diseases, National Institute of Allergy and Infectious Diseases, National Institutes of Health, Bethesda, MD, United States of America; 3 RML Genomics Unit, National Institute of Allergy and Infectious Diseases, National Institutes of Health, Hamilton, MT, United States of America; 4 Applied and Developmental Research Directorate, AIDS Monitoring Laboratory, Leidos Biomedical Research, Inc, Frederick, MD, United States of America; University of Wisconsin, UNITED STATES

## Abstract

Inflammasome-derived cytokines, IL-1β and IL-18, and complement cascade have been independently implicated in the pathogenesis of tuberculosis (TB)-immune reconstitution inflammatory syndrome (TB-IRIS), a complication affecting HIV^+^ individuals starting antiretroviral therapy (ART). Although sublytic deposition of the membrane attack complex (MAC) has been shown to promote NLRP3 inflammasome activation, it is unknown whether these pathways may cooperatively contribute to TB-IRIS. To evaluate the activation of inflammasome, peripheral blood mononuclear cells (PBMCs) from HIV-TB co-infected patients prior to ART and at the IRIS or equivalent timepoint were incubated with a probe used to assess active caspase-1/4/5 followed by screening of ASC (apoptosis-associated speck-like protein containing a CARD domain) specks as a readout of inflammasome activation by imaging flow cytometry. We found higher numbers of monocytes showing spontaneous caspase-1/4/5^+^ASC-speck formation in TB-IRIS compared to TB non-IRIS patients. Moreover, numbers of caspase-1/4/5^+^ASC-speck^+^ monocytes positively correlated with IL-1β/IL-18 plasma levels. Besides increased systemic levels of C1q and C5a, TB-IRIS patients also showed elevated C1q and C3 deposition on monocyte cell surface, suggesting aberrant classical complement activation. A clustering tSNE analysis revealed TB-IRIS patients are enriched in a CD14^high^CD16^-^ monocyte population that undergoes MAC deposition and caspase-1/4/5 activation compared to TB non-IRIS patients, suggesting complement-associated inflammasome activation during IRIS events. Accordingly, PBMCs from patients were more sensitive to *ex-vivo* complement-mediated IL-1β secretion than healthy control cells in a NLRP3-dependent manner. Therefore, our data suggest complement-associated inflammasome activation may fuel the dysregulated TB-IRIS systemic inflammatory cascade and targeting this pathway may represent a novel therapeutic approach for IRIS or related inflammatory syndromes.

## Author summary

Tuberculosis (TB) associated-immune reconstitution inflammatory syndrome (TB-IRIS) is a clinical complication affecting HIV^+^ individuals previously co-infected with *Mycobacterium tuberculosis* (Mtb), upon antiretroviral therapy (ART) initiation. TB-IRIS is characterized by an exacerbated inflammatory response and can be associated with high morbidity and mortality rates in resource-limited countries with high TB prevalence. So far, there is no targeted TB-IRIS therapy, and corticosteroids are frequently used to prevent or alleviate IRIS related-symptoms. Here we found inflammasome activation (i.e. caspase1/4/5^+^ASC speck complex formation) on circulating classical CD14^high^CD16^-^ monocytes may contribute to TB-IRIS immunopathology, since it correlates with pro-inflammatory cytokine plasma levels and its decay is associated with dampening in IRIS-related symptoms promoted by anti-inflammatory therapy. We also found TB-IRIS monocytes display higher surface complement deposition, being more sensitive to external complement-mediated NLRP3 inflammasome activation than healthy control cells. In fact, complement MAC molecule C9 and caspase-1/4/5 activation were associated on classical monocytes in TB-IRIS patients, suggesting complement-mediated inflammasome activation may lead to a positive feedback loop in the inflammatory responses observed in TB-IRIS. Therefore, our findings support that complement-NLRP3/ASC/caspase1/4/5 axis may be considered as a potential target for host-directed therapy of TB-IRIS.

## Introduction

Immune reconstitution inflammatory syndrome (IRIS) is a paradoxical worsening of clinical status experienced by some HIV-infected individuals within a few weeks following antiretroviral therapy (ART) initiation [1–3]. The major feature of IRIS is an exacerbated inflammatory response against the previously associated opportunistic infection, among them Tuberculosis (TB) being the most prevalent [4,5]. TB-IRIS symptoms may vary from fever and lymph node enlargement to sepsis-like syndrome and neurological complications. Therefore, TB-IRIS has emerged as an important clinical issue with high morbidity and mortality rates, especially in resource-limited countries with high TB prevalence [6–9].

A number of innate immune pro-inflammatory cytokines have been implicated in TB-IRIS immunopathogenesis, including IL-6, IL-1β and IL-18 [10–13]. IL-1β and IL-18 production by myeloid lineage cells is a tightly regulated phenomenon relying on inflammasome activation. Canonical inflammasome complexes are pro-caspase-1-activating platforms formed by a cytosolic innate immune sensor and the adapter molecule, ASC (apoptosis-associated speck-like protein containing a caspase activating and recruitment domain (CARD) [14,15]. Upon triggering of inflammasome sensors, ASC recruits pro-caspase-1, which undergoes autoproteolysis becoming an active enzyme. Caspase-1 enzymatic activity is required for IL-1β/IL-18 generation and secretion [16,17] as well as for gasdermin D (GSDMD) cleavage. Cleaved GSDMD forms pores on the plasma membrane of the cells, leading to a necrotic cell death named pyroptosis [18].

Elevated mRNA expression of the pro-inflammatory caspases-1 and 5 and distinct inflammasome sensors, such as absent in melanoma 2 (AIM2) and NLRP3 (NOD-, LRR- and pyrin domain-containing protein 3) have been reported in monocytes from TB-IRIS patients in comparison with TB Non-IRIS post-ART [19,20]. Enhanced active caspase-1 levels in monocytes was also reported during IRIS events, suggesting a role for inflammasomes in TB-IRIS immunopathogenesis [19]. The mechanisms underlying inflammasome activation, however, are still unknown.

Some inflammasome sensors such as the neuronal apoptosis inhibitory protein (NAIP)/NLR family caspase activation and recruitment domain–containing protein 4 (NLRC4) inflammasome [21] and AIM2 [22] detect the presence of either microbial components or host-derived molecules inside the cell cytosol, while NLRP3 seems to be activated through distinct pathways, such as mitochondrial dysfunction [23,24], lysosomal damage [25] and K^+^ efflux [26] that can be elicited by a number of distinct stimuli. More recently, the complement cascade has also been implicated in inflammasome activation [27–30]. It has been shown that signaling pathways downstream of C5aR and C3aR, both receptors for the complement-generated anaphylatoxins, potentiate NLRP3 inflammasome activation and IL-1β secretion by monocytes [27,28]. Moreover, membrane attack complex (MAC, C5b-9) deposition was shown to directly activate the inflammasome [29,30]. It has been proposed that small or sublytic MAC-formed membrane pores allow K^+^ efflux, thus triggering NLRP3 activation.

Interestingly, plasma levels of the classical complement sentinel C1Q were shown to be elevated in TB-IRIS patients compared to controls, suggesting complement activation during IRIS events [31]. Based on these findings, we investigated whether inappropriate complement activity on circulating blood monocytes could contribute to systemic inflammasome activation during IRIS events. Our findings have implications for the management of TB-IRIS and provide insights into the pathogenesis of other infectious or non-infectious inflammatory disorders where inflammasome and complement pathways might play an important role.

## Results

### Inflammasome activation characterizes TB-IRIS events

**Fig 1:**
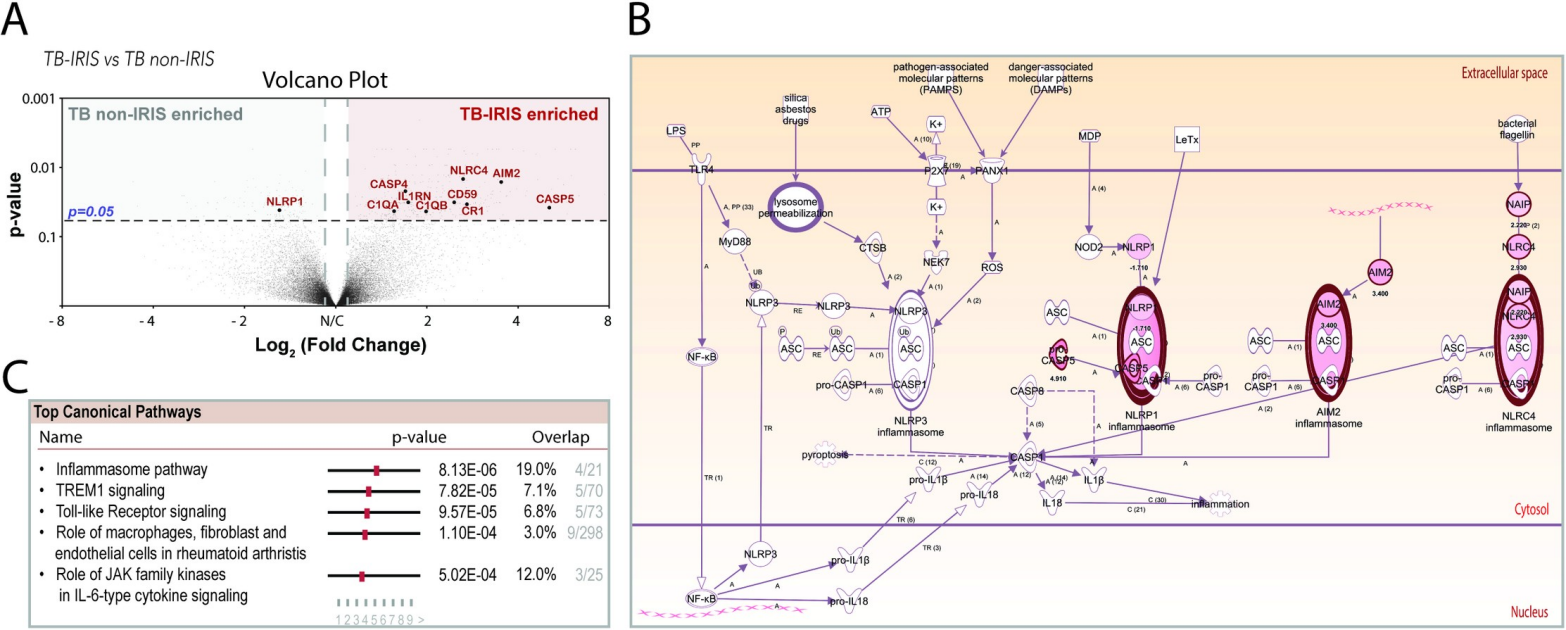
Transcriptional profile of peripheral blood from TB-IRIS and TB non-IRIS patients after ART. (A) Volcano plot of differentially expressed genes in TB-IRIS patients compared to TB-HIV co-infected patients who did not develop IRIS. The top differentially upregulated (log2 fold change 1.5, p = 0.05) genes related to complement and inflammasome pathways are highlighted. (B) Molecule activity prediction was performed using ingenuity pathway analysis (IPA) to predict downstream effects. Molecules in red were those found to be differentially abundant in the signatures of TB-IRIS patients when compared to TB non-IRIS participants. Molecules in light pink were predicted to be activated and arrows show predicted relationships that lead to activation. (C) Canonical pathways significantly differentially regulated are listed according to their p value, the regulation z-score algorithm to identify pathways that are upregulated (positive z-score).

TB-IRIS has been shown to be associated with a wide range of pro-inflammatory cytokines, among them IL-1β and IL-18, known to be driven by inflammasomes. We have performed an RNA microarray analysis in peripheral blood from TB/HIV co-infected patients that had been on TB treatment and ART (characteristics of the subjects in this cross-sectional study cohort, NCT01611402, are summarized in **S1 Table**). We found that patients experiencing IRIS had 167 differentially expressed transcripts compared with matched TB non-IRIS patients (≥ ± 2fold difference, using threshold of p<0.05). We found 19 down and 148 up regulated genes and among them, three inflammasome sensors: AIM2, NAIP5 and NLRC4 as well as the inflammatory caspase-5 (**Fig 1A and 1B**). This finding was further supported by Ingenuity Pathway Analysis (IPA) showing that inflammasome was the top upregulated pathway (p = 4.27e6) during TB-IRIS events, followed by other innate antigen recognition signaling pathways (**Fig 1C**).

**Fig 2:**
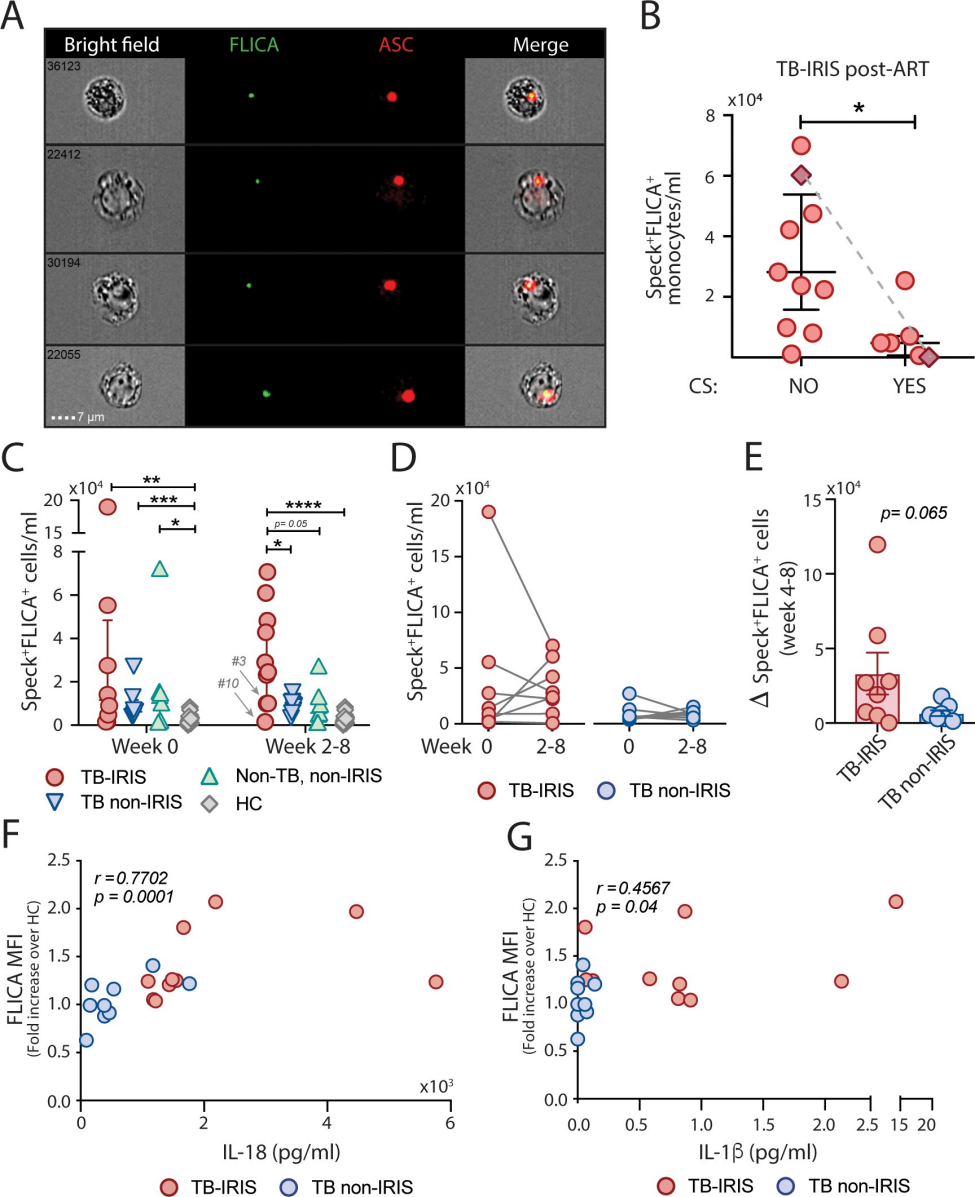
Elevated numbers of monocytes containing FLICA^+^ASC-speck formation is found during TB-IRIS events. (**A**) PBMCs from healthy donors (n = 22) and TB-IRIS (n = 14), TB-non IRIS (n = 9) and Non-TB, non-IRIS (n = 7) patients, before (Week 0) or after ART initiation (Week 2–8), were incubated with the fluorochrome inhibitor of caspase-1/4/5 (FLICA), stained for monocyte identification and intracellular ASC and acquired by using Imaging flow cytometry. Representative images showing co-localization of active caspase-1/4/5 with ASC Specks were selected from a TB-IRIS patient, after Bright Detail Similarity R3_MC_11-ASC_2-FLICA was applied inside the Speck^+^ gate. Images show respectively: BF (brightfield), FLICA and ASC fluorescences followed by a composite image containing BF and the fluorescence of ASC and FLICA merged. Note: The fluorescence intensity of the images may have been modified, without affecting results and statistics. (**B** and **C**) The number of monocytes showing spontaneous FLICA^+^ASC-Speck formation was quantified after application of Modulation_Morphology (M11,Ch11)_11-ASC feature, followed by Bright Detail Similarity R3_MC_11-ASC_2-FLICA, inside the “Total Monocytes” gate, by using IDEAS software. Data are presented as median with interquartile range. *P < 0.05, **P < 0.01 and ****P < 0.001, when Mann-Whitney test was applied. (**D**) Longitudinal analysis of pre-ART *versus* post-ART timepoints for the amount of FLICA^+^ASC-Speck^+^ monocytes/mL found in TB-IRIS (left, n = 8) and TB non-IRIS (right, n = 8) patients. (**E**) Absolute differences (Δ) between pre-ART *versus* post-ART timepoints were calculated for TB-IRIS and TB non-IRIS patient samples. Spearman correlations between plasma levels of IL-18 or IL-1β and caspase-1/4/5 activity (**F and G**, respectively) found on TB-IRIS and TB non-IRIS patient samples. CS = corticosteroids.

To validate the activation of inflammasome during TB-IRIS revealed by transcriptome whole blood analysis, we screened for active ASC aggregates in circulating blood monocytes by imaging flow cytometry [32]. PBMCs from TB-IRIS (n = 14) and TB non-IRIS patients (n = 9) or 7 non-TB HIV^+^ patients (non-TB, non-IRIS), at baseline and after ART initiation (2–8 weeks), as well as healthy controls (HC, n = 22) were pre-incubated with a fluorochrome inhibitor of caspase-1/4/5 (FLICA) followed by immunophenotyping and intracellular ASC staining. Study participants were followed prospectively in three NIH clinical protocols (refer to Methods) and their characteristics are available in **S2 Table**. Gating strategies for the imaging flow cytometry analysis are found in **S1 Fig**. By using this technique, we were able to identify monocytes with cytosolic ASC speck formation associated with active caspase-1/4/5 directly ex vivo in patient samples, without any further stimulation, represented by FLICA^+^ASC-speck^+^ cells (**Fig 2A**, Merge panel).

In absence of a targeted therapy for IRIS, many patients receive nonsteroidal anti-inflammatory drugs (NSAID) or corticosteroids (CS) to control clinical manifestations. To evaluate whether anti-inflammatory therapy may affect our analysis, we compared the inflammasome activation levels post-ART, between TB-IRIS patients treated with CS varying from 3 days to 2 months and patients not receiving CS, even if NSAID were prescribed on an as needed basis (see details in **S2 Table**). Of note, some TB-IRIS patients that had received CS before the studied timepoint were considered off steroids for this analysis. We found that CS treatment was associated with a significantly lower number of FLICA^+^ASC-speck^+^ monocytes (**Fig 2B**). Such effect was better detailed in one patient who was sampled longitudinally (Patient #7 in S2 Table, before and after 1 month of CS), in whom a drastic reduction of FLICA^+^ASC-speck^+^ monocytes was noted after CS treatment (**Fig 2B**, connected diamond symbols). Given the effect of CS therapy on inflammasome assessment, we excluded samples collected during courses of CS from our analysis throughout this work. **Table 1** summarizes the characteristics of the patients analyzed in the remainder of the study.

**Table 1:** Participant characteristics (% or median values with IQR in parenthesis).

	TB-IRIS	TB non-IRIS	Non-TB, non-IRIS	P Value (TB IRIS *vs* TB non-IRIS)	P Value (TB IRIS *vs* non-TB, non-IRIS)
Number of patients	10	9	7		
Male, n (%)	3 (30%)	6 (66.67%)	3 (42.86%)	0.110	0.585
Age	35.5 (33.0–43.0)	38.0 (35.5–49.0)	29.0 (22.0–32.0)	0.174	**0.006**
Black race, n (%)	9 (90%)	7 (77.7%)	4 (57.1%)	0.460	0.110
Time between start of TB treatment and ART	27.00 (12.75–33.25)	28.00 (19.50–29.00)	N/A	0.888	N/A
Time on antiretroviral therapy	4 weeks (2–4)	4 weeks (3–4)	4 weeks (2–4)	0.633	0.733
CD4^+^ T-cell count at study entry, cells/μL	39.50 (25.50–71.50)	31.50 (17.75–71.00)	60.00 (14.00–726.0)	0.585	0.459
CD4^+^ T-cell count at post-ART timepoint, cells/μL	133.5 (72.25–263.8)	55.00 (34.00–109.0)	115.0 (54.00–611.0)	**0.022**	0.600
HIV viral load at study entry, log_10_ copies/mL, x 10^3^	509 (112–962)	70.0 (16.35–210.3)	15.8 (4.0–43.9)	**0.008**	**0.009**
HIV viral load at post-ART timepoint, log_10_ copies/mL	480.5 (224.0–4157)	208.0 (74.75–267.3)	75.00 (49.00–316.0)	0.056	**0.042**
ALC*/μL	927.0 (360.6–1122)	1057 (722.2–1474)	992 (714–1874)	0.277	0.314
AMC*/μL	443.2 (267.3–797.5)	220.3 (223.5–438.5)	322.4 (190.8–539.7)	0.277	0.269
ANC*, x 10^3^/μL	5.5 (3.5–6.9)	1.9 (1.4–2.9)	2.3 (1.8–3.2)	**0.001**	**0.043**

* ALC = Absolute leukocyte count; AMC = Absolute monocyte count; ANC = Absolute neutrophil count. **P Value** when Mann-Whitney or Chi-square, df was applied.

At the pre-ART timepoint, the three groups of HIV^+^ patients had significantly higher numbers of monocytes with FLICA^+^ASC-speck^+^ formation when compared with the HC group (**Fig 2C, week 0**), consistent with previous reports showing HIV-induced inflammasome activation in viremic patients [33,34] without statistically significant differences between the groups although some patients who ultimately developed TB-IRIS following ART had high pre-ART levels of FLICA^+^ASC-speck^+^ monocytes (**Fig 2C, week 0**). On the other hand, TB-IRIS patients not receiving anti-inflammatory treatment at their respective IRIS timepoints (n = 10), exhibited significantly higher numbers of monocytes with FLICA^+^ASC-speck^+^ formation when compared to TB non-IRIS group (n = 9) and non-TB, non-IRIS (n = 7) groups at matched timepoints or HCs (TB-IRIS median: 25,952 monocytes/ml [IQR: 9,128–50,665] *vs* TB non-IRIS: 9,200 monocytes/ml [IQR: 6,325–10,872] (**Fig 2C, week 2–8**). Interestingly, 2 patients (Patient #3 and Patient #10) highlighted in **Fig 2C** (week 2–8), with low levels of inflammasome assembly, had received treatment with CS but therapy was completed before sampling (see in details in **S2 Table**), suggesting CS therapy may have a lingering effect on the inflammasome assessment in circulating monocytes.

By comparing patients longitudinally, we observed that some of the TB-IRIS and TB non-IRIS patients experienced a reduction of FLICA^+^ASC-speck^+^ monocytes/ml (**Fig 2D**), consistent with a decline in the viral load promoted by the ART treatment (**Tables 1 and S2**). On the other hand, some patients had an increase in the number of monocytes with inflammasome activation post-ART in both patient groups (**Fig 2D**), suggesting a distinct course between inflammasome and plasma viremia post-ART. Accordingly, pre and post-ART variations on the amount of monocytes with inflammasome assembly and viral loads do not correlate (**S2A Fig**) although viremia might contribute to monocyte priming, therefore affecting the enzymatic activity of caspase-1, measured as the mean fluorescence intensity of the caspase-1 substrate FLICA by flow cytometry (**S2B Fig**). Of note, variations in CD4 counts pre and post-ART timepoints did not correlate with inflammasome activation in patient samples (**S2C and S2D Fig**). Interestingly, absolute changes in the number of monocytes showing inflammasome assembly between pre-ART *versus* post-ART timepoints were more robust in the TB-IRIS group than in the TB non-IRIS group **(Fig 2E)**. Independently of the direction of these changes, however, TB-IRIS patients showed sustained higher levels of inflammasome activation post-ART (**Fig 2C and 2D**). Accordingly, we found that plasma levels of IL-18 and IL-1β, which have been found to be elevated in TB-IRIS compared to TB non-IRIS individuals [10–13], correlated with caspase-1/4/5 activity levels (**Fig 2F and 2G**, respectively).

**Fig 3:**
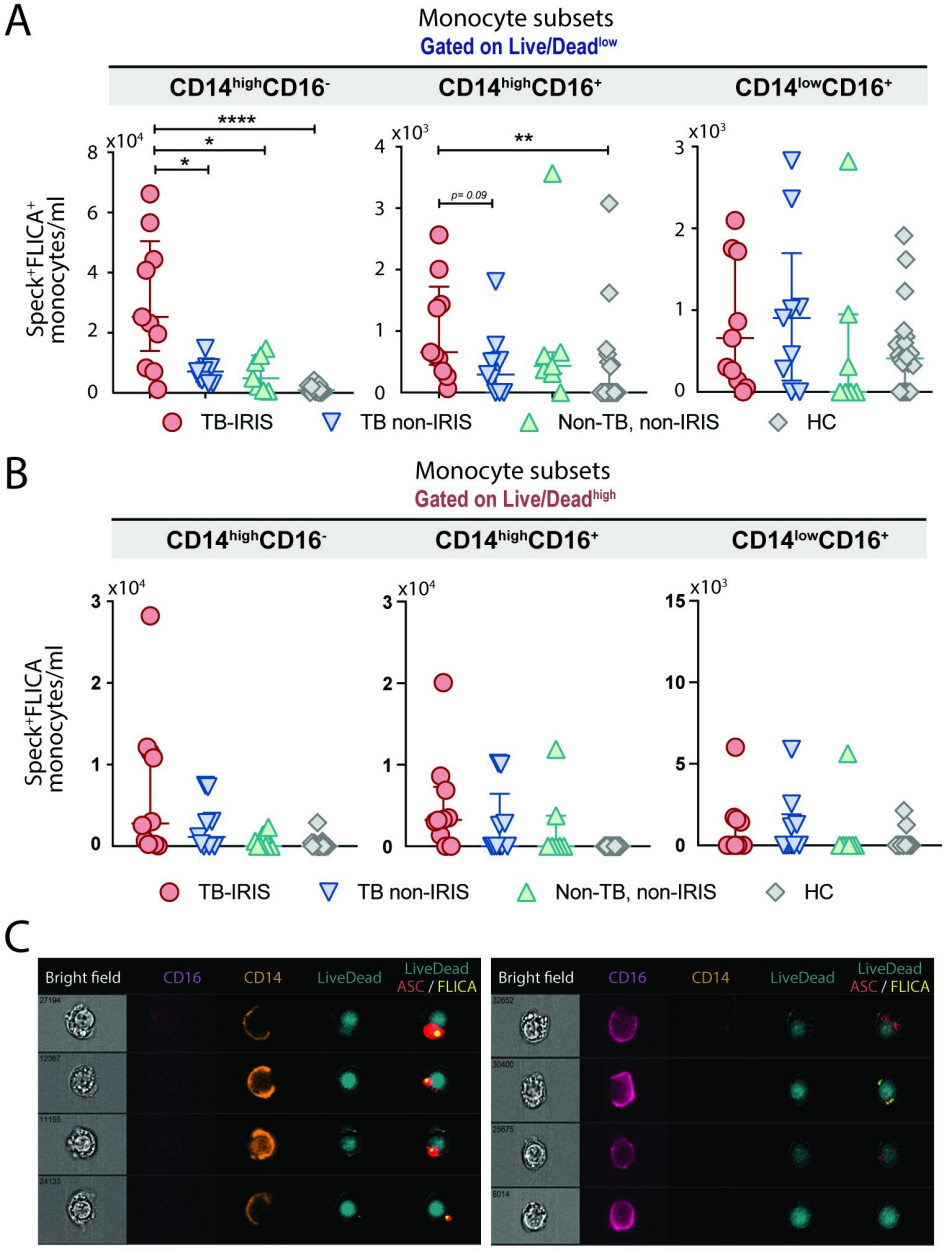
Quantification of canonical inflammasome activation within distinct monocyte subsets in different stages of pyroptosis. FLICA^+^ASC-speck^+^ monocytes/ml were quantified in PBMCs from healthy volunteers and patients after modulation_morphology (M11,Ch11),11-ASC, followed by bright detail similarity R3_MC_11-ASC_2-FLICA features were applied in all data points, inside Live/Dead AQUA^low^ (**A**) or Live/Dead AQUA^high^ (**B**) gates. *P < 0.05, **P < 0.01 and ****P < 0.001, when Mann-Whitney test was applied. (**C**) Representative images of FLICA^+^ASC-speck^+^ monocytes inside the Live/Dead AQUA^high^ gate within CD14^high^CD16^-^ subset (left panel) or CD14^low^CD16^+^ subset (right panel) were selected, showing respectively: BF1 (brightfield), CD16, CD14, Live/Dead (L/D) and a composite image containing the fluorescence of Live/Dead AQUA, FLICA and ASC.

Since we have previously reported that the expansion of the CD14^high^CD16^-^ classical monocyte subset is associated with the occurrence of TB-IRIS following ART [11], we analyzed each of the major monocyte subsets (i.e. classical—CD14^high^CD16^-^, Intermediated—CD14^high^CD16^+^ and non-classical or patrolling—CD14^low^CD16^+^) for inflammasome activation. In fact, we observed that the classical CD14^high^CD16^-^ subset is responsible for the higher amount of FLICA^+^ASC-speck^+^ in total monocytes observed in TB-IRIS patients (**Fig 3A**). Of note, although the inflammasome complex can be released to the extracellular milieu at the late stages of pyroptosis [35,36], we also found FLICA^+^ASC-speck^+^ monocytes within the Live/Dead^high^ gate (**Fig 3B**), representing cells in early stages of pyroptotic cell death. Again, most of these cells corresponded to the CD14^high^CD16^-^ subset, as shown in **Fig 3C,** where we can observe membrane “breaks”, stained by the CD14 marker versus dying cells with an intact plasmatic membrane seen by the CD16 staining. Collectively, our data show that ASC-speck formation on CD14^high^CD16^-^ monocytes, a canonical inflammasome activation hallmark, is specifically associated with TB-IRIS clinical events following ART.

### TB-IRIS patients show prominent complement activity during IRIS events

**Fig 4:**
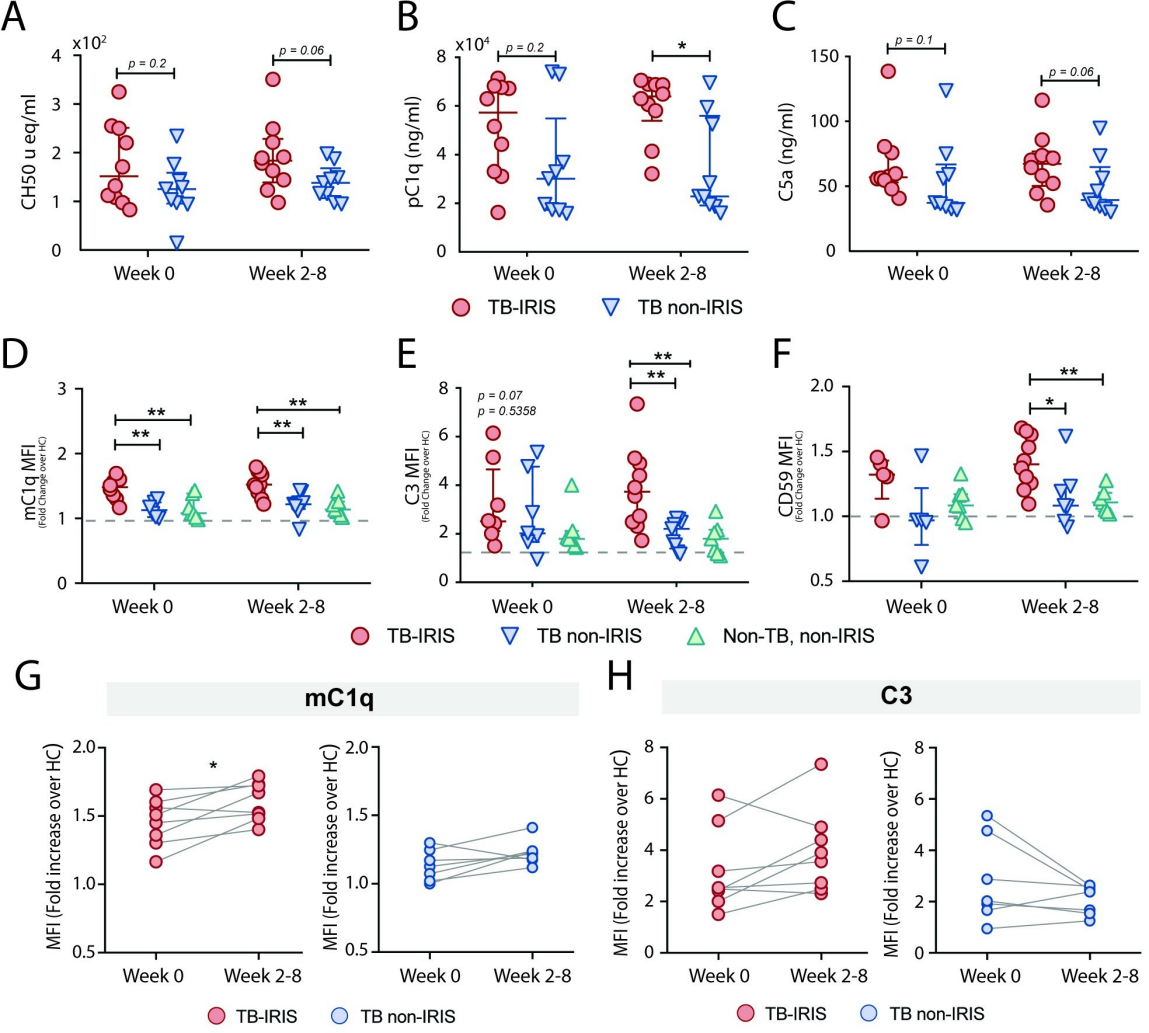
TB-IRIS is accompanied by complement overactivation. (**A**) Serum or (**B**)(**C**) plasma samples from TB-IRIS and TB non-IRIS patients, before (Week 0) or after ART initiation (Week 2–8), were collected and tested for classical complement activity (**A**), C1q (**B**) or C5a quantification (**C**). Membrane-bound C1q (mC1q) (**D**), C3 (**E**) and CD59 (**F**) expression levels were quantified on total monocytes from TB-IRIS, TB non-IRIS and Non-TB, non-IRIS patients, by flow cytometry. Expression levels (MFIs) were calculated as fold change over their experimental respective HCs. Data are presented as median with interquartile range. *P < 0.05 and **P < 0.01, when Mann-Whitney test was applied. Longitudinal analysis of pre-ART *versus* post-ART timepoints for mC1q (**G**) and C3 (**H**) expression levels for TB-IRIS (n = 8, left side) and TB non-IRIS (n = 7, right side) patients. *P < 0.05 was considered statistically significant when Wilcoxon signed-rank test was applied.

The complement cascade actively regulates some components of the inflammatory response and has also been implicated in TB-immune reconstitution inflammatory syndrome [37]. Consistently, our transcriptome analysis revealed that C1q, the complement receptor CR1 and the membrane complement inhibitor CD59, were upregulated in TB-IRIS individuals in comparison with those who did not develop IRIS, post-ART (**Fig 1A**). To assess complement activity in TB-IRIS events, we measured CH50 in sera of patients. This assay measures the ability of a serum to lyse antibody-sensitized sheep erythrocytes via classical complement pathway-mediated membrane attack complex (MAC) formation [38]. The CH50 assay demonstrated enhanced classical complement activity in plasma from TB-IRIS individuals compared to controls, at post-ART timepoints, although the difference was not statistically significant (p = 0.06, **Fig 4A**). Accordingly, significantly higher plasma levels of C1q (pC1q - **Fig 4B**) were found in TB-IRIS in comparison with TB non-IRIS patients post-ART. Moreover, plasma levels of the complement anaphylatoxin C5a was also elevated in TB-IRIS in comparison with TB non-IRIS patients post-ART (p = 0.06). Collectively, these findings suggest activation of the classical complement pathway in TB-IRIS events.

Although primarily targeting pathogen cell surfaces, activation of complement may overcome protective complement regulators and may thus affect bystander cells and result in host tissue damage [39]. We further characterized the activation of the complement cascade in TB-IRIS by measuring the membrane-bound complement components on circulating monocytes. We found that TB-IRIS participants had increased levels of monocyte membrane-bound C1q (mC1q) (**Fig 4D**) and C3 (mC3) (**Fig 4E**) when compared to TB non-IRIS and non-TB, non-IRIS groups, post-ART (mC1q: TB-IRIS, median of 1.521 fold change [IQR: 1.376–1.724] *vs* TB non-IRIS 1.219 [IQR: 1.147–1.326] and mC3: TB-IRIS, median of 3.730 fold change [IQR: 2.445–4.952] *vs* TB non-IRIS 2.203 [IQR: 1.396–2.535]).

To counterbalance undesired effects of complement activation, multiple complement regulators are distributed as either soluble effectors or cell surface-bound inhibitors, such as CD59, which binds to the C5b-8 complex, and represents the major cell surface inhibitor of full MAC assembly (reviewed in Refs [39,40]). In fact, along with C1q, we demonstrated that CD59 was also transcriptionally upregulated in PBMCs from IRIS individuals at the IRIS event timepoint (**Fig 1A**). Accordingly, we found elevated CD59 surface expression levels in TB-IRIS patients when compared to control groups (**Fig 4F)**, suggestive of a compensatory mechanism in the context of a persistently activated complement cascade. Interestingly, except for mC1q, which is shown to be broadly distributed among the distinct monocyte subgroups (**S3A Fig**), both mC3 and CD59 upregulation seem to follow the inflammasome pattern, and were expressed more prominently within the CD14^high^ subsets of monocytes (**S3B and S3C Fig**, respectively).

Furthermore, we observed that complement deposition on monocytes from patients who developed IRIS tends to be enhanced at IRIS events when compared to pre-ART timepoints, reaching statistical significance for membrane-bound C1q (**Fig 4G and 4H**). In contrast, most of the TB non-IRIS patients seem to not significantly increase mC1q (**Fig 4G**) or even decrease the C3 surface expression levels, post-ART (**Fig 4H**). Taken together, these findings demonstrate that TB-IRIS is accompanied by enhanced classical complement activity, resulting in complement deposition on the cell surface of monocytes with some possibly compensatory upregulation of CD59 on CD14^high^ monocytes.

### Inflammasome overactivation is associated with membrane attack complex (MAC) deposition in TB-IRIS patients

**Fig 5:**
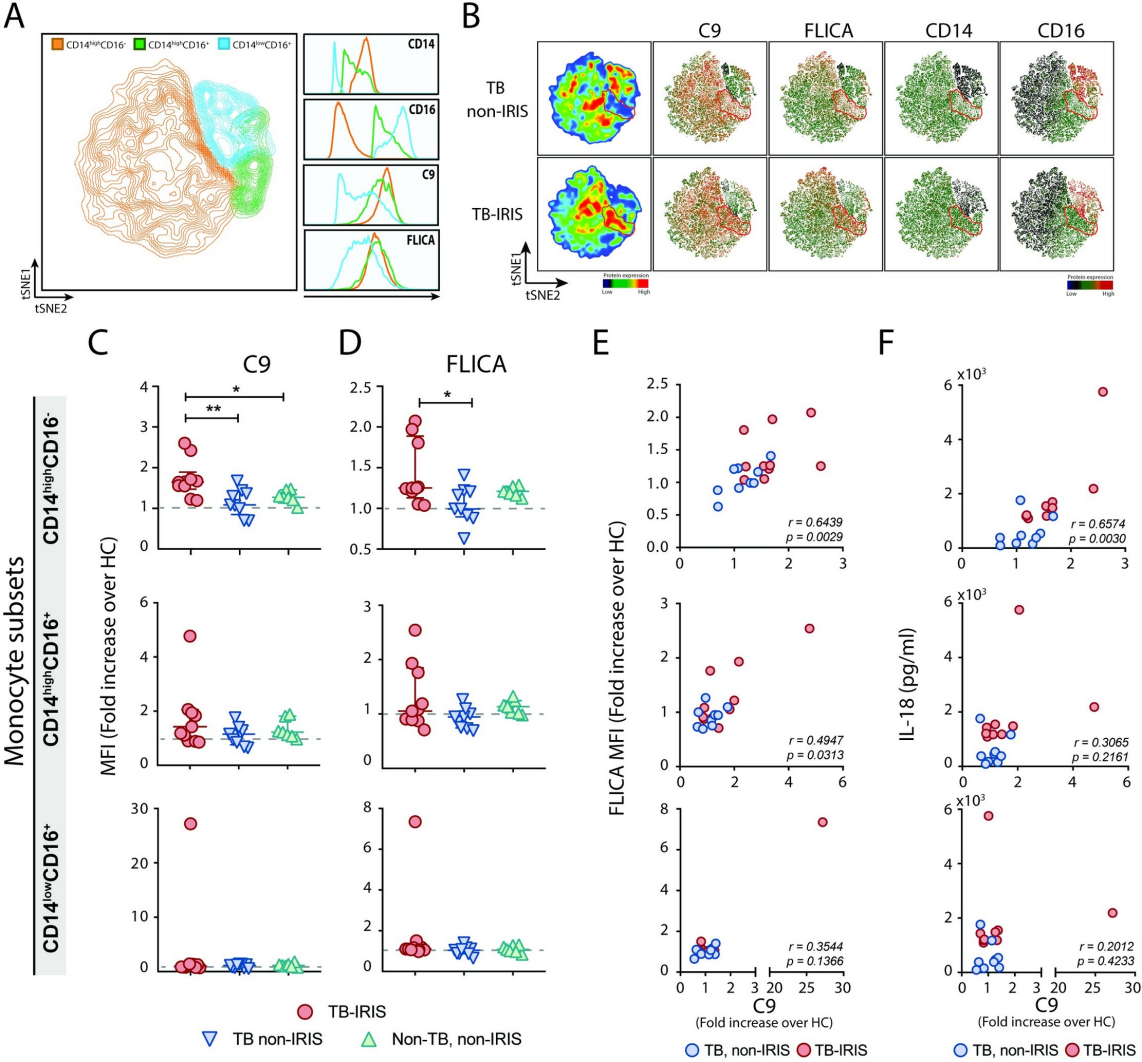
Association between complement MAC deposition and inflammasome overactivation on monocytes during IRIS event. **(A)** Overlay of monocyte subsets clusters from HLADR^+^DUMP^-^CD14^+^ and CD16^+^ population (“Total monocytes”) of TB-IRIS (n = 3) and TB non-IRIS (n = 3) patients on top of the t-SNE map based on CD14, CD16, C9 and FLICA expression. **(A–right panel)** Representative histograms of CD14, CD16, C9 and FLICA expression levels in each monocyte subset presented in the t-SNE plot. **(B)** Individual t-SNE maps for each group of samples (TB-IRIS and TB non-IRIS) based on expression levels of C9, FLICA, CD14 and CD16. Circles are drawn to highlight cell populations discussed in the text. **(C)** C9 and **(D)** FLICA expression levels (MFIs) were calculated as fold change over their respective experimental HCs for TB-IRIS (n = 10), TB non-IRIS (n = 9) and Non-TB, non-IRIS patients (n = 7), post-ART, within the distinct indicated monocytes subsets. Data are presented as median with interquartile range. *P < 0.05, **P < 0.01, when Mann-Whitney test was applied. Spearman correlations between FLICA fold change levels **(E)** or plasma levels of IL-18 **(F)** and C9 fold change levels found on monocytes from TB-IRIS and TB non-IRIS patient samples.

Since both complement and inflammasome pathways were found to be activated during TB-IRIS, we next explored a possible cooperative interaction between these regulators of the inflammatory responses in monocytes from TB-HIV patients. To address this issue, we incubated TB-IRIS (n = 3) and TB non-IRIS (n = 3) PBMCs in their IRIS or matched timepoints with the active caspase-1/4/5 probe (FLICA) followed by extracellular C9 staining for MAC measurement. We then performed a t-Distributed Stochastic Neighbor Embedding (tSNE) analysis on a panel including the two monocyte phenotypic markers (CD14 and CD16) besides C9 and FLICA, where TB-IRIS and TB non-IRIS monocytes were directly compared and clustered against each other (for more details, please refer to the method section). By gating on each monocyte subset according to CD14 and CD16 expression and then overlaying those events on top of the tSNE plot, we can observe the contribution of each subset to the overall structure of the population (**Fig 5A**). Comparing the expression levels of C9 and FLICA within the monocyte subsets, we found that CD14^high^ monocytes (classical and intermediated groups) express higher levels of C9 and active caspase-1/4/5 in comparison with the non-classical/patrolling subset (**Fig 5A**–right panels).

By evaluating the contribution of each group of patient samples (TB non-IRIS and TB-IRIS) to the overall structure of the tSNE plot, we observed that TB-IRIS patients are enriched in a population corresponding to the CD14^high^ monocytes (**Fig 5B**–within red circled area), while the CD14^low^CD16^-^ group was greatly reduced in comparison with TB non-IRIS controls (**Fig 5B**), thus showing that TB-IRIS samples were the major contributors for the CD14^high^ monocyte clusters in the overall structure of the tSNE plot shown in **Fig 5A**. Indeed, we observed that the CD14^high^ monocyte population that emerged in TB-IRIS patients had high MAC and FLICA expression (red clusters within red circled area), suggesting that TB-IRIS patients were enriched in a CD14^high^ monocyte population with complement deposition and caspase-1 activation. Summary data of C9 and FLICA fold-change MFIs in patient samples over their respective HC values confirm that patients experiencing TB-IRIS had higher levels of C9 deposition (**Fig 5C**) associated to increased caspase-1/4/5 activation (**Fig 5D**) compared to matched controls. Also, differences among groups were better observed within the CD14^high^CD16^-^ subset and were progressively less noticeable in intermediate and patrolling subsets, respectively. Consistently, C9 deposition in classical CD14^high^CD16^-^ monocytes is significantly upregulated following ART when compared with pre-ART levels in patients who developed TB-IRIS, but not in non-IRIS patients when analyzed longitudinally (**S4A Fig**). Of note, variations in HIV viral loads or CD4 counts pre *versus* post-ART timepoints did not seem to affect complement C9 deposition in patient samples (**S4B** and **S4C Fig**, respectively).

Finally, consistent with a cooperative interaction between complement and inflammasome activation during the IRIS events, C9 and FLICA expression levels positively correlated within the CD14^high^CD16^-^ subset (**Fig 5E**). C9 expression also correlates with plasma levels of IL-18 (**Fig 5F**), in the same monocyte subset. These data suggest sublytic MAC-driven inflammasome activation on CD14^high^ monocytes may represent an amplification mechanism contributing to the inflammatory “cytokine storm” characterizing IRIS.

### PBMCs from TB-HIV patients are more prone to complement-mediated NLRP3-dependent IL-1β secretion

Our data suggest a crosstalk between complement MAC and caspase-1/4/5 activation during IRIS events. The activation of inflammasomes as a result of sublytic MAC deposition, however, has only been described so far in murine bone marrow–derived dendritic cells and human lung epithelial cells or THP-1 lineage [28–30]. To evaluate a direct role of complement in inflammasome activation in circulating human monocytes, we sensitized column-purified fresh isolated HC monocytes with anti-CD59, and then stimulated cells with different concentrations of rabbit serum (RS), as a source of complement proteins, in order to induce classical complement activity. As a negative control for complement deposition, monocytes were stimulated with heat-inactivated RS (HI-RS).

**Fig 6:**
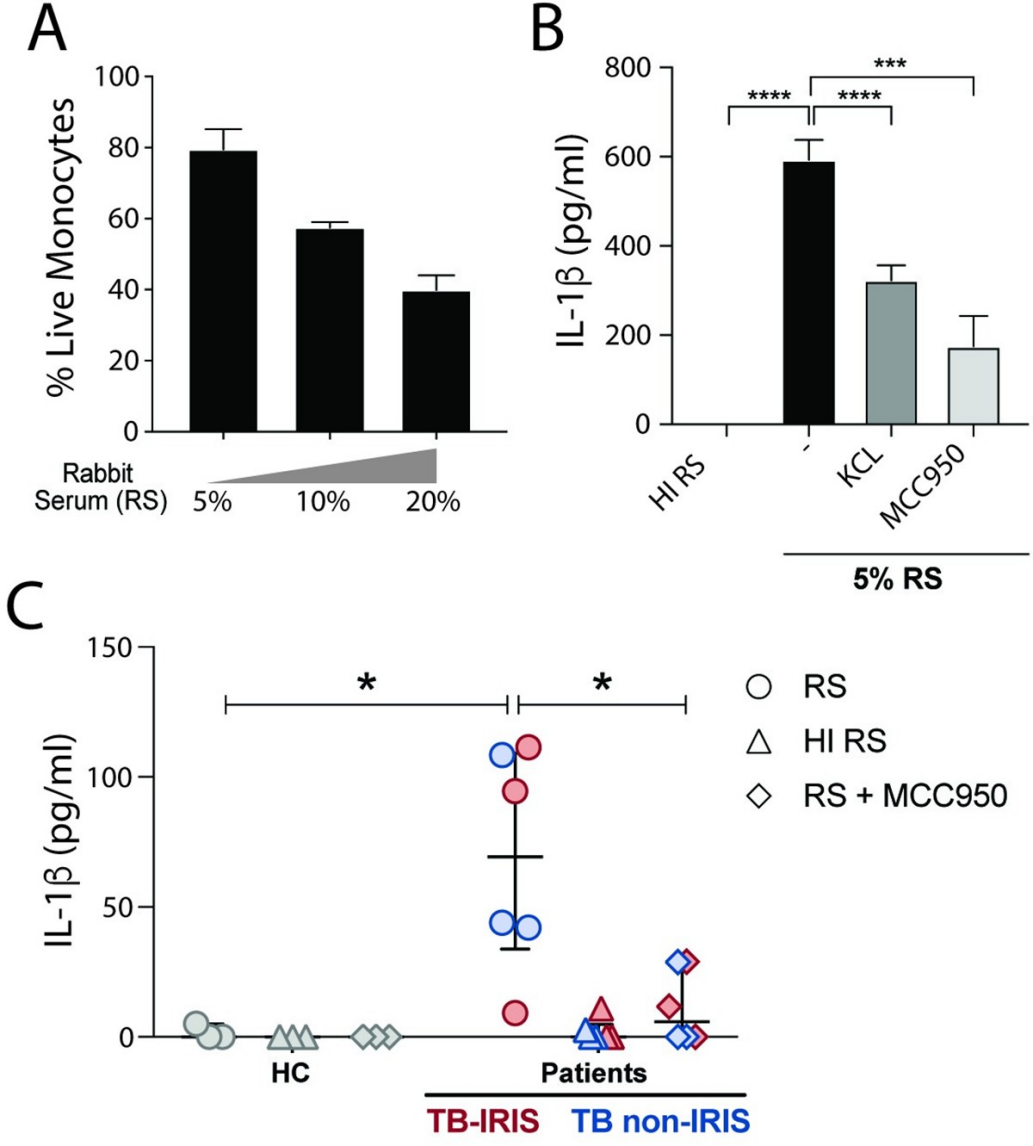
Effect of exogenous complement-mediated inflammasome activation on HCs and patient cells. **(A)** Absolute numbers of healthy control human monocytes stimulated with designated concentrations of rabbit serum (RS) or heat-inactivated (HI)-RS (30 min at 56°C) for 2 hours, were enumerated by using counting beads by flow cytometry. HI-RS-stimulated cultures were considered 100% viable. **(B)** IL-1β secretion of 5% RS or 5% HI-RS-stimulated healthy control monocytes was determined at culture supernatants by ELISA. Cells were treated or not with the NLRP3 inhibitor, MCC950 (3 μM) or with KCl (10 mM) solution. Numbers represent the means ± SEM (*n* = 3). ****P < 0.001 when compared with control or untreated groups. Data are representative of three independent experiments. **(C)** IL-1β secretion by PBMCs from three different HCs, TB non-IRIS or TB-IRIS patients, at IRIS timepoint, was determined by ELISA after cells were incubated or not with MCC950 (3 μM) and exposed to 3% of RS or HI-RS for 2 hr. Data are presented as median with interquartile range. *P < 0.05 when compared with control or untreated groups after Mann-Whitney test was applied.

RS induced a concentration-dependent cell death, consistent with complement-dependent cytotoxicity (CDC) (**Fig 6A**). In order to assess inflammasome activation triggered by sublytic complement deposition, we used RS or HI-RS at the lower concentration (5%) and assessed IL-1β secretion in culture supernatants. We found that RS, but not HI-RS supplementation resulted in IL-1β secretion (**Fig 6B**), thus confirming complement-dependent inflammasome activation in monocytes.

Sublytic MAC assembly on plasma membrane results in K^+^ efflux from cytoplasm to the extracellular *milieu*, in turn, the lower intracellular concentration of K^+^ triggers the NLRP3-mediated inflammasome activation [29,30]. Interestingly, KCl supplementation in cell culture media by reducing the gradient driving the osmotic K^+^ efflux, results in inhibition of RS-induced IL-1β secretion (**Fig 6B**). Moreover, IL-1β release is drastically reduced by MCC950, a selective inhibitor of NLRP3 (**Fig 6B**). Of note, either NLRP3 inhibitor or KCl specifically inhibited IL-1β secretion by PAM3CSK4-primed and nigericin-stimulated monocytes (**S5A and S5B Fig**), without affecting TNF-α secretion (**S5C and S5D Fig**).

We further tested whether patient cells are more sensitive to *ex-vivo* complement-mediated inflammasome activation when compared to HC cells. PBMCs from three distinct TB-IRIS and TB-non-IRIS patients and three HCs were thawed and incubated at a low concentration of RS or HI-RS (3%) with or without the NLRP3 inhibitor MCC950. We observed higher IL-1β production from patient cells in comparison with HCs, which was abrogated in response to HI-RS (**Fig 6C**). Moreover, RS-induced IL-1β production was strongly dampened upon NLRP3-inflammasome inhibition (**Fig 6C**).

Thus, our findings demonstrate that inflammasome activation in complement-stimulated human monocytes is mediated by K^+^ efflux/NLRP3-dependent mechanism. In addition, PBMCs obtained from patients are more prone to inflammasome activation triggered by exogenous source of complement than cells isolated from HC individuals.

## Discussion

Complement and inflammasome are important antimicrobial and inflammation-generating arms of the innate immune system [41–43]. These molecular pathways, however, can be harmful when dysregulated, and have been independently implicated in several autoimmune and chronic inflammatory diseases [44–55]. Herein, we show that overactivation of both complement system and inflammasome pathways are associated with paradoxical TB-IRIS events. Although the increased activity of caspase-1/4/5 in monocytes from TB-IRIS patients in comparison with non-IRIS patients has been previously reported [19], to our knowledge, the present study is the first to report the association between active caspase-1/4/5 and complement cascade in patient monocytes, thereby outlining a mechanism involved in spreading inflammation observed in TB-IRIS.

Our transcriptome analysis revealed that TB-IRIS events are characterized by transcriptional upregulation of innate immune signaling pathways, in accordance with previous reports [19,20]. Of note, lymphocyte and monocyte counts were not significantly different between TB-IRIS and TB non-IRIS patients included in the microarray analysis (**S1 Table**). Although neutrophil counts were shown to be significantly different between the groups tested, we previously showed that the plasma inflammatory profile of TB-IRIS patients is mainly driven by monocyte activation, and is not associated with absolute counts of monocytes or neutrophils in the peripheral blood [11].

We found that TB-IRIS patients exhibit upregulation of AIM2, NAIP5 and NLRC4 inflammasome mRNA compared to controls, therefore complementing the transcriptional profiling of a previous report which documented AIM2 and NLRP3 upregulation in isolated monocytes from TB-IRIS patients [19]. Supporting a role for NLRP3 inflammasome sensor in TB-IRIS events, we observed that complement-driven IL-1β production by patient PBMCs was drastically reduced in the presence of the NLRP3 inhibitor MCC950. Of note, it is possible that inflammasome sensors other than NLRP3 are also involved with TB-IRIS immunopathology. In fact, it is reasonable to consider the sensing of bacterial DNA and/or self-DNA derived from mitochondrial stress or cell death in TB-IRIS events, mediated by the AIM2 inflammasome [56]. Moreover, a common *NLRC4* gene polymorphism has been recently associated with modified systemic and pulmonary inflammation in TB-HIV co-infected patients [57], revealing new roles for NLRC4 in addition to its function as the NAIP5 inflammasome adapter in response to flagellated bacteria products [21].

ASC-speck formation is widely considered a hallmark of canonical inflammasome activation [58], since it promotes pro-caspase-1 autoproteolysis thus allowing the cleavage of pro-IL-1β and pro-IL-18 into their mature forms [17]. We found elevated numbers of FLICA^+^ASC-speck^+^ CD14^high^CD16^-^ monocytes in TB-IRIS subjects compared to TB non-IRIS controls, further supporting aberrant inflammasome activation as a key event in the immunopathogenesis of IRIS. Also, the presence of FLICA^+^ASC-speck^+^ monocytes within the Live/Dead^high^ gate suggests cells undergoing pyroptosis. Along with intracellular inflammatory contents, pyroptotic cells have been shown to release ASC aggregates to the extracellular space at late stages of cell death [35,36]. Free ASC particles act as “damage signals” either directly targeting extracellular pro-caspase-1 and/or being engulfed by neighboring cells thus activating NLRP3 through lysosomal disruption. In that sense, free ASC-specks may contribute to spread inflammation during IRIS. Accordingly, it was shown that pyroptotic classical monocytes from HIV-1-infected patients release ASC complex into the blood stream, which may contribute to immune activation during the course of HIV infection [33].

Along with disseminated inflammasome activity, our CH50 data also demonstrate that TB-IRIS clinical events are accompanied by systemic activation of the classical complement cascade which is triggered by soluble or membrane-bound Antigen-Antibody complex that binds C1q [59]. Accordingly, we also found increased protein levels of C1q and C3 on monocytes cell surface, suggesting complement activation on host cells during TB-IRIS events. In fact, undesired complement activity on bystander host cells have been reported in acute pathologic conditions, such as sepsis, or chronic diseases, due to an ongoing inflammatory process, the presence of immune complexes, and/or apoptotic/necrotic cells [39]. Furthermore, it has been shown that IFN-γ-activated circulating blood monocytes and tissue macrophages are major sources of C1q [60] and the intrinsic C1 complex (C1q, C1s, C1r) can be placed on monocyte surface thus triggering full activation of the complement cascade [61]. Given that we have found higher expression of C1q mRNA in PBMCs from TB-IRIS patients than controls, our findings also support a role for intrinsic C1q-driven complement activation on monocytes during TB-IRIS, in addition to external C1q deposition.

One possible explanation for the emergence of the complement signaling pathway upon ART-driven immune reconstitution in TB-IRIS patients may be attributed to the enhanced expansion of Mtb-specific effector memory CD4^+^ T lymphocytes in comparison with TB non-IRIS patients [62–64]. The encounter of these Th1 lymphocytes, which are a significant source of TNF-α and IFN-γ, with TB-primed innate immune cells can induce the generation of inflammatory cytokines and complement components by the innate immune cells [39,60]. In turn, these inflammatory mediators, including the anaphylatoxins C3a and C5a, also promote transcriptional upregulation of the inflammasome sensors and pro-IL-1/IL-18, a process called inflammasome signal 1 [65,66]. Therefore, reconstitution of the Mtb-specific CD4^+^ T cells could support paradoxical complement activity and inflammasome activation following ART, even after reduction of HIV viral load. It is not clear, however, whether pre-ART higher TB antigen load within innate immune cells is underlying the exacerbated expansion of the Mtb-specific CD4^+^ T cells in patients who develop IRIS.

Furthermore, complement activity on host cells surface may result in complement-dependent cytotoxicity (CDC) through full MAC deposition or in sublytic MAC-driven NLRP3 inflammasome activation [37,67]. Accordingly, we found enrichment of a population of classical monocytes showing C9 deposition and caspase-1/4/5 activity in TB-IRIS patients compared to patients who do not experience IRIS. In addition, patient PBMCs were more prone than HC cells to activate NLRP3 and secrete IL-1β in response to a low concentration external source of complement. This enhanced sensitivity observed on patient cells may be attributed to a pre-activated state due to the presence of the initial steps of the complement cascade as well as upregulated inflammasome components. Together, these data suggest complement cascade can be upstream of inflammasome activation during IRIS events, and thus fueling the systemic inflammatory response observed in TB-IRIS. In agreement with that, it has been shown that complement pathway gene activation is one of the earliest events of disease in HIV-TB co-infected patients followed by the activation of the inflammasome gene pathway post-ART [68].

Our data are corroborated by previous findings showing the classical CD14^high^CD16^-^ monocyte subset is expanded in TB-IRIS patients in contrast with the CD14^low^CD16^-^ patrolling group, and the frequency of those cells correlates with IL-1β plasma levels during IRIS events [11]. Moreover, we found that the complement cell-associated inhibitor CD59 followed the C9 and FLICA expression pattern and was upregulated in the CD14^high^ subsets. Given that CD59 is known to block the formation of large lytic MAC pores in an attempt to avoid CDC, but still allows for assembly of sublytic MAC [69, 70], it is possible that upregulation of CD59 by a group of classical/Intermediated monocytes could render these cells less sensitive to CDC and therefore more prone to sublytic MAC-induced inflammasome activation than patrolling monocytes. In addition, the loss of the CD14^high^ monocytes (either by CDC or pyroptosis) could be preventing their differentiation into the CD14^low^ subset [71,72]. Both possibilities would result in the shrinkage of the patrolling group, a feature observed in TB-IRIS patients. Further investigation is needed to elucidate whether distinct monocyte subsets present intrinsically different responses to complement and/or inflammasome activation.

The emergence of the complement-associated inflammasome signaling pathway after ART initiation may contribute to TB-IRIS immunopathology, since it correlates with IL-1β and IL-18 plasma levels and its decay is associated with dampening in IRIS-related symptoms promoted by anti-inflammatory therapy. Consistently, crosstalk between MAC and inflammasome activation was recently reported as the mechanism underlying inflammation during complement-mediated autoinflammatory diseases like paroxysmal nocturnal hemoglobinuria [73] and rheumatoid arthritis [74]. Therefore, our findings suggest the use of drugs designed to inhibit either the complement cascade or the NLRP3/ASC/caspase-1/4/5 molecules as a potential new therapeutic approach to treat TB-IRIS.

## Methods

### Ethics statement

Study participants were followed prospectively in three NIH clinical protocols: Immune Reconstitution Syndrome in HIV-Infected Patients Taking Antiretroviral Therapy (IRIS), NCT00286767; Gene Expression in HIV and Tuberculosis co-infection (TSHAI), NCT01611402 and PET Imaging and Lymph Node Assessment of IRIS in People with AIDS (PANDORA), NCT02147405. IRIS and PANDORA were evaluating people with HIV (PWH) who were either ART-naïve with CD4 counts<100 cells/μL and starting ART or PWH on ART evaluated with symptoms of IRIS. Healthy volunteer blood samples were obtained on an NCI, NIH IRB-approved protocol 99-CC-0168 and were de-identified prior to distribution. All participants provided written informed consent in accordance with the Declaration of Helsinki.

### Plasma biomarker measurements

Cryopreserved plasma samples from patients were assessed to measure levels of IL-18 by electrochemiluminescence (Meso SCALE, Gaithersburg, MD), IL-1β (R&D Systems, Minneapolis, MN), C1q and C5a (ABCAM, Cambridge, MA) by ELISA and CH50 by EIA (MicroVue Eq total classical complement activity assay from Quidel San Diego, CA) following the manufacturer’s instructions.

### Microarray analysis

Blood was collected in PAXgene tubes at the time of TB-IRIS or, for those without IRIS, at 8–12 weeks after ART initiation, then cryopreserved. RNA was extracted and hybridized on the Affymetrix GeneChip Human Gene 2.0 ST array. The chip was then stained and scanned. Expression of transcripts was compared using ANOVA with multiple test correction using Benjamini–Hochberg false discovery rate method. Ingenuity Pathway Analysis (IPA) was used to identify canonical pathways and upstream regulators.

### Cell culture and treatments

Cryopreserved ficoll-isolated peripheral blood mononuclear cells (PBMCs) from patients or healthy control individuals (HCs) were thawed and resuspended in RPMI-1640 media (Corning, NY) supplemented with 10% heat-inactivated human AB serum (Gemini Bio-Products, West Sacramento) and 0.05% benzonase (MilliporeSigma, Massachusetts). Cells were rested for 1 hour at 37°C and 5% CO_2_ and subsequently plated at 10^6^ cells/well in round bottom 96-well plates (Corning Costar, MilliporeSigma) followed by immune staining. To achieve canonical NLRP3 inflammasome activation as a positive control, HC PBMCs were first primed with the TLR2 ligand, Pam3CSK4 (Invivogen, San Diego, California) (0.5 μg/mL, 2 hr) and further stimulated with 3.5 μM of Nigericin (1hr) (Invivogen) to promote signal two for NLRP3 inflammasome activation. Cells were incubated in the presence or absence of the selective NLRP3 inhibitor MCC950 from Invivogen (0.5, 1 or 3 μM) or KCl solution (5, 10 or 30 mM) to prevent K^+^ efflux. IL-1β and TNF-α were quantified in culture supernatants by using Multi-Analyte Flow Assay Kit (LEGENDplex) from Biolegend, following the manufacturer’s instructions.

### Complement deposition assay

As a positive control for complement-mediated inflammasome activation, circulating blood monocytes were isolated from fresh HC PBMCs by using the EasySep Human Monocyte Enrichment Kit without CD16 depletion (Stemcell technologies) and plated at 2 x 10^5^ cells/well. Cells were sensitized with mouse polyclonal anti-human CD59 (BRIC 229, from NHS Blood and Transplant, North Bristol Park Filton Bristol BS34 7QH UK), for 30 min at room temperature (RT). Cells were then exposed to different percentages of rabbit serum (RS) (Cedarlane, Burlington, NC) or heat-inactivated (HI)-RS (30 min at 56°C), at 37°C for 2 hours and absolute cell numbers were enumerated by flow cytometry, by using counting beads (ThermoFisher, Rockford, IL). Alternatively, cells were exposed to 5% RS or HI-RS in the presence or absence of MCC950 (3 μM) or KCl (10 mM) for inflammasome inhibition. For comparisons with patient cells, cryopreserved PBMCs from HCs or TB-IRIS and TB non-IRIS patients were thawed and incubated with anti-human CD59 (30 min, RT) followed by stimulation with 3% RS or HI-RS, in the presence or absence of MCC950 (3 μM). Supernatants were collected for IL-1β measurement by ELISA.

### Inflammasome assessment by imaging flow cytometry

Inflammasome complex assembly was evaluated by detection of ASC speck formation by imaging flow cytometry, as previously described [32]. Briefly, cells were incubated with the fluorochrome inhibitor of caspase-1/4/5 (FLICA, from Immunochemistry technologies (ICT), Bloomington, MN) following the manufacturer’s instructions, for 50 min at 37°C, to allow for binding of FLICA to activated inflammatory caspases. Cells were washed twice with FLICA wash buffer and then incubated with LIVE/DEAD Fixable AQUA Dead Cells Stain (Thermo Fisher, Massachusetts) for 15 min at RT, followed by extracellular staining in PBS + 1% BSA with fluorochrome-conjugated antibodies for monocyte phenotyping as listed in **S3 Table**. Cells were fixed and permeabilized with Cytofix/Cytoperm (BD Biosciences) for 1h at RT and stained with anti-ASC/TMS1 antibody from Novus Biologicals, CO, USA, for another 1 hr. Samples were washed twice and resuspended in 50 μL of PBS+1%BSA.

### Image acquisition and analysis

Approximately 40,000 focused cells for each sample were acquired using a 12 channel Amnis ImageStreamX Mark II (MilliporeSigma) imaging flow cytometer equipped with the 405 nm, 488 nm and 642 nm lasers. Channel 2 (FLICA-AF488), Channel 3 (DUMP = CD2, CD3, CD19, CD20, CD56, CD66b –PE), Channel 7 (HLA-DR), Channel 8 (Live/Dead-AQUA), Channel 10 (CD14-BV605) and Channel 11 (ASC-AF647) were selected. For brightfield and SSC detection (785nm laser), channels 1/9 and 12 were used, respectively. Samples were acquired at 60X magnification and low speed/high resolution. The integrated software INSPIRE (MilliporeSigma) was used for data collection. Images were analyzed using image-based algorithms in the ImageStream Data Exploration and Analysis Software (IDEAS 6.2.64.0, MilliporeSigma) as described previously [32].Gating strategies are listed in **S1 Fig**.

### Flow cytometry

Immunophenotyping of patients and healthy control PBMCs was performed by flow cytometry. Cells were stained with 2 distinct panels of antibodies prepared in Gelatin Veronal Buffer (GVB) (CompTech complement Technology Inc, Tyler, TX) for 30 min at room temperature (RT) to characterize complement and caspase-1 activation in monocytes. The panels with antibody clones and fluorochromes are listed in **S3 Table**. Staining protocol and analysis of monocytes proceeded as described above for imaging flow cytometry analysis. Data were acquired on a BD LSR II flow cytometer (BD Biosciences). All compensation and gating analyses were performed using FlowJo 10.5.3 (TreeStar, Ashland, OR). For t-Distributed Stochastic Neighbor Embedding (tSNE) analysis, gated monocytes (“total monocytes” defined as (DUMP^-^(CD2, CD3, CD19, CD20, CD56, CD66b) HLADR^+^ population) from TB-IRIS (n = 3) and TB non-IRIS (n = 3) patient samples were downsampled to create separate subgroups containing an equal number of events (31,500 events/monocytes per subgroup) by using FlowJo 10.6.1. Subgroups were then electronically concatenated and annotated with identifying keywords. tSNE analysis was then performed as follow: perplexity = 20, iterations = 550 and rate = 4410. Compensated data from the same experiment and day of sample acquisition were used for analysis.

### Statistical analyses

Statistical analyses were performed using non-parametric Mann-Whitney test in GraphPad Prism 4 software (GraphPad, USA). Data are presented as median with interquartile ranges. Differences between groups were considered significant when *p* < 0.05.

## Supporting information

S1 Fig Gating strategies for identification of FLICA^+^ASC-speck^+^ human circulating blood monocytes. (**A**) Single cells were gated from cell aggregates and debris by using a scatter plot of the brightfield of area *versus* aspect ratio. Next, a histogram of gradient RMS of the brightfield channel (channel 1) identified cells in best focus. From the focused single cell population, a classical flow cytometry dot-plot of Area versus Side Scatter (SSC) was applied to identify, based on size and granularity, myeloid-like cells from the PBMC suspension. Monocytes were further defined as HLADR^+^ and DUMP (CD2, CD3, CD19, CD20, CD56, CD66b)^-^ cells. Three major monocyte subsets were considered for analysis based on CD14 and CD16 surface expression: classical/inflammatory (CD14^high^CD16^-^), intermediate (CD14^high^CD16^+^) and patrolling (CD14^low^CD16^+^) monocytes. Differential analysis based on Live/Dead expression were made on total monocytes or inside each subset gate. Positive cells for ASC and FLICA were gated for downstream analysis of the ASC expression pattern. The “Modulation” feature was used as the strategy for ASC-specks identification. We then applied the default application wizard of the IDEAS software “co-localization”, that measures the co-localization of two probes with punctate staining by adding a histogram of bright detail similarity R3 for the double positive population in the analysis area (here, ASC and FLICA) to identify FLICA^+^ASC-speck^+^ cells.
(TIF)

S2 Fig Correlations between inflammasome activation levels and clinical parameters. Spearman correlations between absolute differences (Δ) of the amount of FLICA^+^ASC-speck^+^ monocytes or caspase-1/4/5 activity levels and HIV viral load **(A and B)** or CD4 counts **(C and D)** pre-ART *versus* post-ART timepoints.
(TIF)

S3 Fig Complement deposition within distinct monocyte subsets. **(A)** mC1q, **(B)** C3 and **(C)** CD59 expression levels (MFIs) were calculated as fold change over their respective experimental HCs for TB-IRIS (n = 10) and TB non-IRIS (n = 9) patients, post-ART, within the distinct indicated monocytes subsets. Data are presented as median with interquartile range. *P < 0.05 and **P < 0.01 when Mann-Whitney test was applied.
(TIF)

S4 Fig Longitudinal analysis and correlations with clinical factors for C9 deposition within the classical CD14^high^CD16^-^ monocyte subset. **(A)** Longitudinal analysis of pre-ART *versus* post-ART timepoints for C9 expression levels for TB-IRIS (n = 8) and TB non-IRIS (n = 8) patients. **P < 0.01 was considered statistically significant when Wilcoxon signed-rank test was applied. Spearman correlations between absolute differences (Δ) of C9 deposition and Viral loads **(B)** or CD4 counts **(C)** pre-ART *versus* post-ART timepoints.
(TIF)

S5 Fig Effects of MCC950 and KCl on cytokine secretion from pam3CSK4-primed and nigericin-stimulated cells. (**A** and **B**) IL-1β or (**C** and **D**) TNFα levels from supernatants of healthy control PBMCs primed with the TLR2 ligand, Pam3CSK4 (0.5 μg/mL, 2 hr) and further stimulated with 3.5 μM of Nigericin (1hr) were determined by multi-analyte flow assay kit. Cells were pretreated or not with the NLRP3 inhibitor, MCC950 (**A** and **C**) or with a KCl solution (**B** and **D**), with the indicated concentrations, 1h prior to stimulation with Nigericin and inhibitors were maintained in cell culture. Numbers represent the means ± SEM (*n* = 3). *P < 0.05; **P < 0.01 when compared with the untreated group.
(TIF)

S1 Table Characteristics of study participants in the transcriptome analysis. (DOCX)

S2 Table Characteristics of study participants. (DOCX)

S3 Table List of antibodies used in ImageStream and flow cytometry panels. (DOC)
