# Supplementary figures and images for: Classical complement and inflammasome activation converge in CD14highCD16- monocytes in HIV associated TB-immune reconstitution inflammatory syndrome
Source: PLoS Pathog. 2021 Mar 31;17(3):e1009435. doi: 10.1371/journal.ppat.1009435 (PMC8041190; doi:10.1371/journal.ppat.1009435)

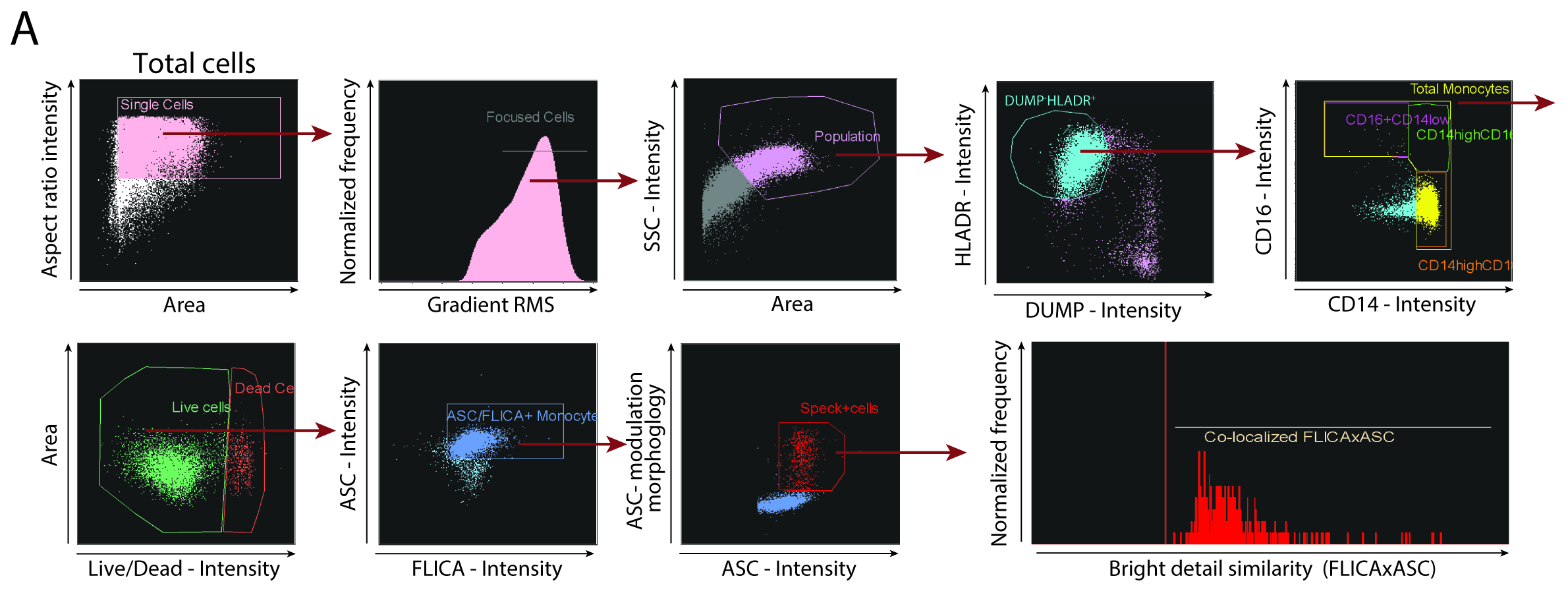

Supplement: S1 Fig — (A) Single cells were gated from cell aggregates and debris by using a scatter plot of the brightfield of area versus aspect ratio. Next, a histogram of gradient RMS of the brightfield channel (channel 1) identified cells in best focus. From the focused single cell population, a classical flow cytometry dot-plot of Area versus Side Scatter (SSC) was applied to identify, based on size and granularity, myeloid-like cells from the PBMC suspension. Monocytes were further defined as HLADR+ and DUMP (CD2, CD3, CD19, CD20, CD56, CD66b)- cells. Three major monocyte subsets were considered for analysis based on CD14 and CD16 surface expression: classical/inflammatory (CD14highCD16-), intermediate (CD14highCD16+) and patrolling (CD14lowCD16+) monocytes. Differential analysis based on Live/Dead expression were made on total monocytes or inside each subset gate. Positive cells for ASC and FLICA were gated for downstream analysis of the ASC expression pattern. The “Modulation” feature was used as the strategy for ASC-specks identification. We then applied the default application wizard of the IDEAS software “co-localization”, that measures the co-localization of two probes with punctate staining by adding a histogram of bright detail similarity R3 for the double positive population in the analysis area (here, ASC and FLICA) to identify FLICA+ASC-speck+ cells. (TIF) [file ppat.1009435.s001.tif]

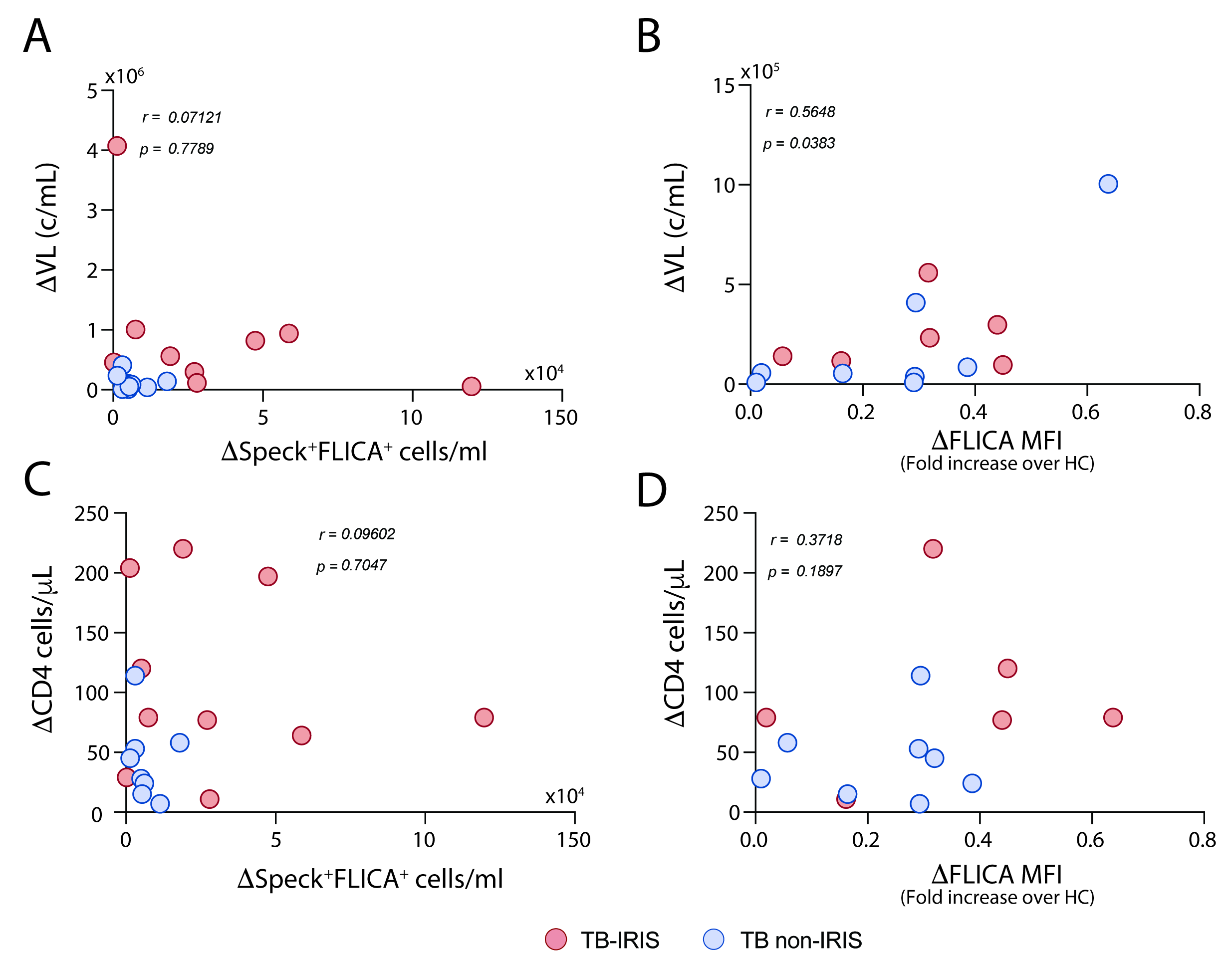

Supplement: S2 Fig — Spearman correlations between absolute differences (Δ) of the amount of FLICA+ASC-speck+ monocytes or caspase-1/4/5 activity levels and HIV viral load (A and B) or CD4 counts (C and D) pre-ART versus post-ART timepoints. (TIF) [file ppat.1009435.s002.tif]

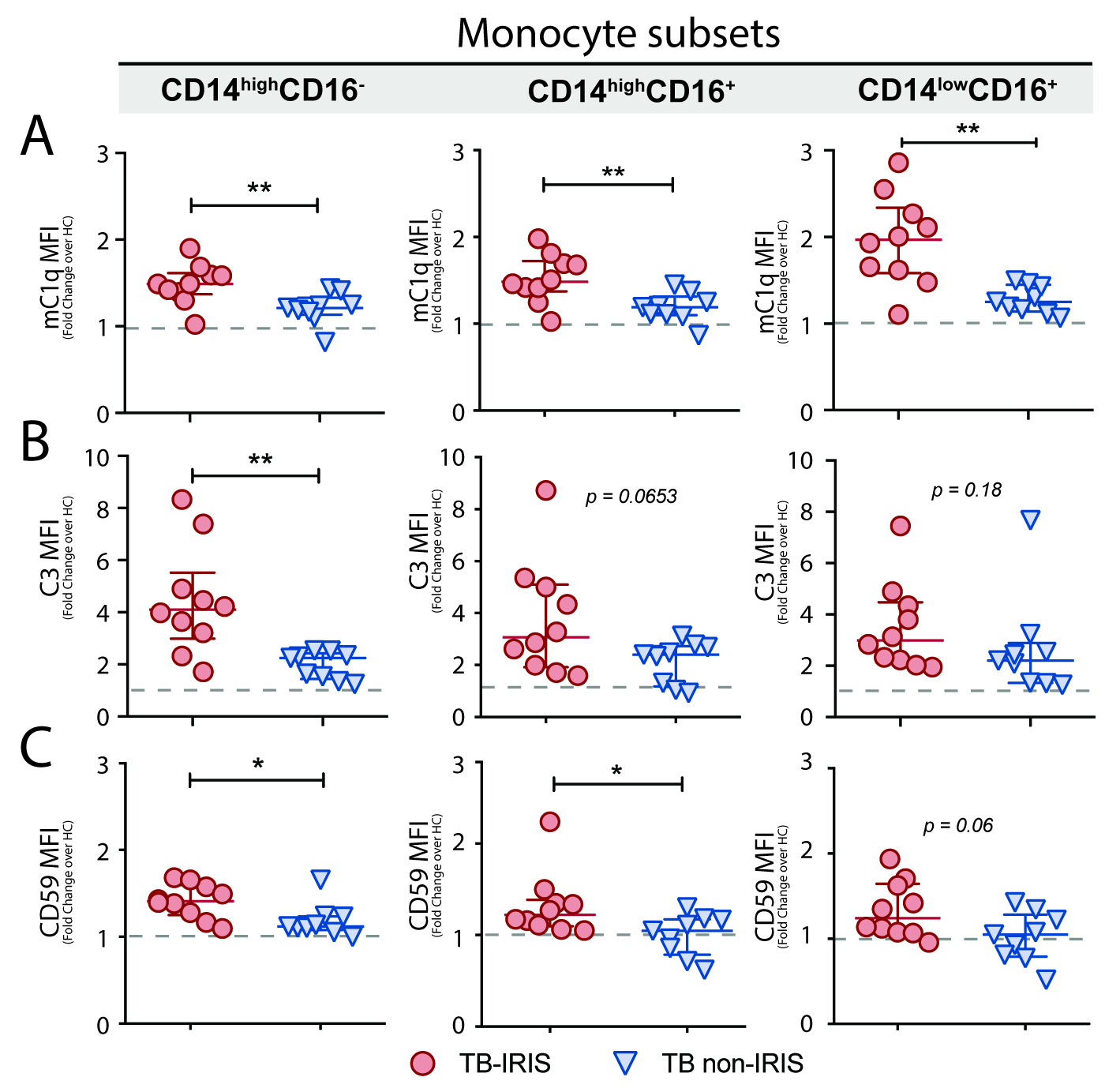

Supplement: S3 Fig — (A) mC1q, (B) C3 and (C) CD59 expression levels (MFIs) were calculated as fold change over their respective experimental HCs for TB-IRIS (n = 10) and TB non-IRIS (n = 9) patients, post-ART, within the distinct indicated monocytes subsets. Data are presented as median with interquartile range. *P < 0.05 and **P < 0.01 when Mann-Whitney test was applied. (TIF) [file ppat.1009435.s003.tif]

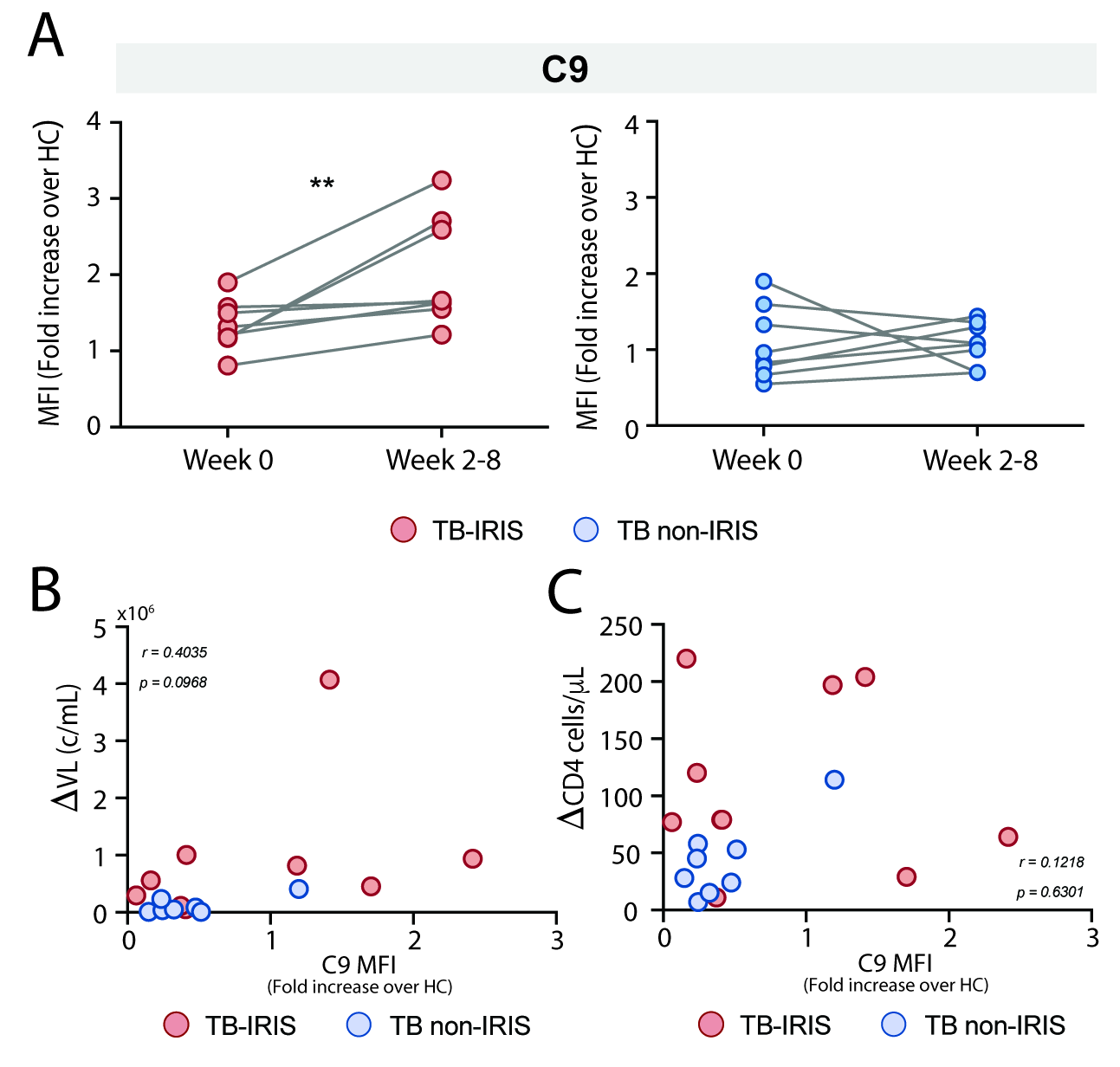

Supplement: S4 Fig — (A) Longitudinal analysis of pre-ART versus post-ART timepoints for C9 expression levels for TB-IRIS (n = 8) and TB non-IRIS (n = 8) patients. **P < 0.01 was considered statistically significant when Wilcoxon signed-rank test was applied. Spearman correlations between absolute differences (Δ) of C9 deposition and Viral loads (B) or CD4 counts (C) pre-ART versus post-ART timepoints. (TIF) [file ppat.1009435.s004.tif]

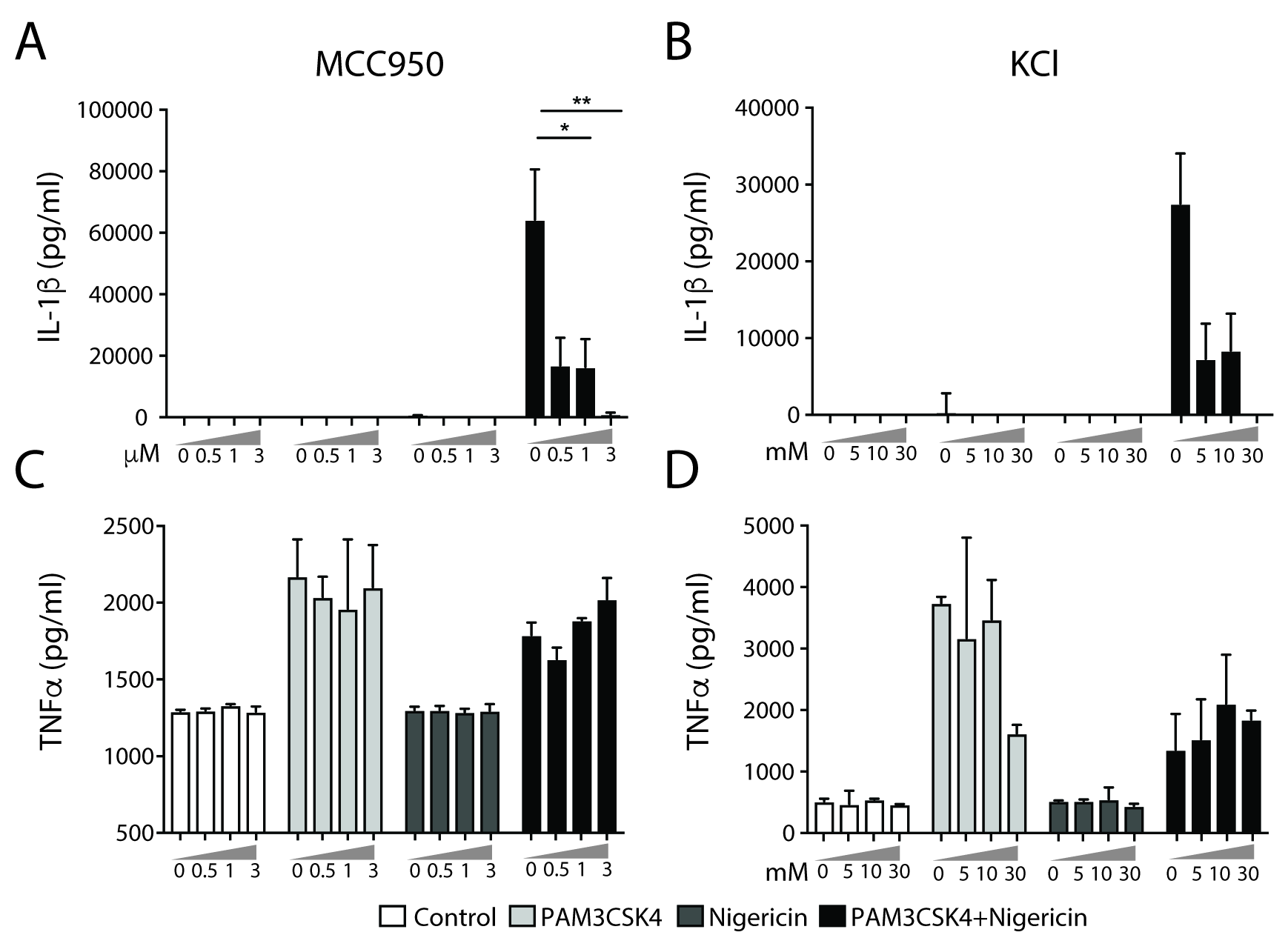

Supplement: S5 Fig — (A and B) IL-1β or (C and D) TNFα levels from supernatants of healthy control PBMCs primed with the TLR2 ligand, Pam3CSK4 (0.5 μg/mL, 2 hr) and further stimulated with 3.5 μM of Nigericin (1hr) were determined by multi-analyte flow assay kit. Cells were pretreated or not with the NLRP3 inhibitor, MCC950 (A and C) or with a KCl solution (B and D), with the indicated concentrations, 1h prior to stimulation with Nigericin and inhibitors were maintained in cell culture. Numbers represent the means ± SEM (n = 3). *P < 0.05; **P < 0.01 when compared with the untreated group. (TIF) [file ppat.1009435.s005.tif]
