# Supplementary material for: Classical complement and inflammasome activation converge in CD14highCD16- monocytes in HIV associated TB-immune reconstitution inflammatory syndrome
Source: PLoS Pathog. 2021 Mar 31;17(3):e1009435. doi: 10.1371/journal.ppat.1009435 (PMC8041190; doi:10.1371/journal.ppat.1009435)
Supplement: S1 Table — (DOCX) [file ppat.1009435.s006.docx]

|  | **TB-IRIS** | **TB non-IRIS** | **P Value** |
| --- | --- | --- | --- |
| Number of patients | 6 | 10 |  |
| Male, n (%) | 2 (33.3%) | 6 (60%) | 0.30 |
| Age, median (IQR) | 35.5 (32.3-43.8) | 37.5 (35.8-43.5) | 0.44 |
| Black race, n (%) | 5 (83.3%) | 6 (60%) | 0.59 |
| Days on antiretroviral therapy, median (IQR) | 12.0 (6.8-23.3) | 60.5 (56-69.8) | **0.001** |
| Time between start of TB treatment and ART, median (IQR) | 37 (31.8-49.3) | 45.5 (30.5-103) | 0.48 |
| CD4^+^ T-cell count at post-ART timepoint tested, cells/µL, median (IQR) | 107 (65.7-183) | 127 (55.5-294) | 0.78 |
| HIV viral load at post-ART timepoint tested, log_10_ copies/mL x 10^3^, median (IQR) | 1.38 (0.075-11.167) | 0.056 (0.039-0.105) | 0.0589 |
| ALC/μL, median (IQR)* | 504.5 (217.1-1279) | 1061 (642.2-1645) | 0.0992 |
| AMC/μL, median (IQR)* | 254.8 (161.3-555.8) | 316.7 (223.5-491.0) | 0.6787 |
| ANC, x 10^3^/μL, median (IQR)* | 4.1 (3.2-9.4) | 1.8 (1.2-2.6) | **<0.001** |

**S1 Table. Characteristics of study participants in the transcriptome analysis.**

* ALC = Absolute leukocyte count; AMC = Absolute monocyte count; ANC = Absolute neutrophil count
