# Supplementary material for: Classical complement and inflammasome activation converge in CD14highCD16- monocytes in HIV associated TB-immune reconstitution inflammatory syndrome
Source: PLoS Pathog. 2021 Mar 31;17(3):e1009435. doi: 10.1371/journal.ppat.1009435 (PMC8041190; doi:10.1371/journal.ppat.1009435)
Supplement: S2 Table — (DOCX) [file ppat.1009435.s007.docx]

**S2 Table. Characteristics of study participants.**

| **TB-IRIS** | **Timepoint** | **CD4 cells/µL (%)** | **VL (c/mL)** | **ARV Regimen** | **Time on TB therapy pre-ART** | **Steroids/immunomodulants at or prior to studied timepoints** | **Co-infections** |
| --- | --- | --- | --- | --- | --- | --- | --- |
| Patient 1 | *Pre-ART* | *5 (2%)* | *56, 824* | Atripla | 48 days | No | TB, Thrush, HPV, Oral hairy leukoplakia, Presumptive MAC |
|  | *Week 8* | *84 (10%)* | *<50* |  |  |  |  |
| Patient 2 | *Pre-ART* | *35 (9%)* | *298,438* | Biktarvy | 46 days | Yes, on naproxen as needed at Week 4 timepoint | TB, Thrush |
|  | *Week 4* | *112 (35%)* | *294* |  |  |  |  |
| Patient 3 | *Pre-ART* | *88 (4%)* | *1,005,306* | RAL/TDF/FTC until Week 7 then DOL/TDF/FTC | 53 days | Yes, on hydrocortisone 20mg daily at pre-ART timepoint with a duration of 18 days of hydrocortisone taper prior to timepoint | TB, KS, HPV |
|  | Week 4 | 154 (7%) | 492 |  |  | Yes, had received hydrocortisone taper that completed 21 days prior to Week 4 timepoint |  |
| Patient 4 | *Pre-ART* | *52 (9%)* | *96,401* | Atripla | 37 days | No | TB, Hep B |
|  | *Week 4* | *155 (13%)* | *134* |  |  |  |  |
| Patient 5 | Week 2 | 263 (26%) | 3,629 | Atripla (+RAL) | 37 days | No | TB, HSV |
| Patient 6 | *Pre-ART* | *32 (3%)* | *116,763* | Atripla | 41 days | Yes, on dexamethasone 1mg daily at Pre-ART timepoint with a dexamethasone taper duration of 7 weeks prior to timepoint | TB, Thrush, HPV, C.diff |
|  | Week 4 | *43 (6%)* | 262 |  |  | Yes, on ibuprofen as needed at Week 4 timepoint |  |
| Patient 7 | *Pre-ART* | *6 (3%)* | 947,162 | Atripla | 25 days | No | TB, Cryptosporidium, Multicentric Castleman's, HSV, Hep B, C. Diff |
|  | Week 1 | 70 (23%) | 5,741 |  |  | No |  |
|  | Week 5 | 36 (6%) | 550 |  |  | Yes, on prednisone 40mg at Week 5 timepoint with a duration of 4 weeks of prednisone taper prior to timepoint |  |
| Patient 8 | *Pre-ART* | *46 (6%)* | *560,924* | DOL/TDF/FTC | 13 days | No | Mycobacterial infection, Thrush, VSV |
|  | Week 3 | 266 (15%) | 1,610 |  |  |  |  |
| Patient 9 | *Pre-ART* | *44 (5%)* | *456,875* | Atripla | 34 days | No | TB, HSV, Thrush |
|  | Week 4 | 73 (18%) | 254 |  |  |  |  |
| Patient 10 | *Pre-ART* | *158 (32%)* | *4,078,372* | RAL/TDF/FTC | 53 days | Yes, on prednisone 5mg daily at Pre-ART timepoint with hydrocortisone taper duration of 6 weeks prior to timepoint | TB, KS, Histoplasmosis, HSV, Thrush |
|  | *Week 2* | *362(46%)* | *7438* |  |  | Yes, had received hydrocortisone/prednisone taper completed 18 days prior to timepoint |  |
| Patient 11 | *Week 12* | *81 (19%)* | *<50* | Atripla | 40 days | Yes, on prednisone 80mg at Week 12 timepoint with a duration of 6 weeks of prednisone prior to timepoint. Also on ibuprofen as need at Week 12 timepoint. | TB, Thrush |
| Patient 12 | Week 2 | 57 (16%) | 201 | RAL/TDF/FTC | -4 days | Yes, on prednisone 50mg at Week 2 timepoint with a duration of 11 days of prednisone taper prior to timepoint | Disseminated TB, KICS, HSV, HPV |
| Patient 13 | *Week 8* | *49 (4%)* | *<40* | DOL/TDF/FTC | 37 days | Yes, on prednisone 40mg at Week 8 timepoint with a duration of 9 days of prednisone taper prior to timepoint | TB, Hep B |
| Patient 14 | *Week 2* | *77 (14%)* | *335* | DOL/TDF/FTC then Triumeq 2 days prior to Week 2 timepoint | 38 days | Yes, on prednisone 40mg at Week 2 timepoint with a duration of 3 days of prednisone taper prior to timepoint | TB, Thrush |

| **TB non-IRIS** | **Timepoint** | **CD4 cells/µL (%)** | **VL(c/mL)** | **ARV Regimen** | **Time on TB Therapy pre-ART** | **Steroids/Immunosuppressant at or prior to studied Timepoints** | **Co-infections** |
| --- | --- | --- | --- | --- | --- | --- | --- |
| Patient 1 | *Pre-ART* | *32 (4%)* | *141,255* | RAL/TDF/FTC | 36 days | No | TB, HSV, Thrush |
|  | *Week 2* | *90 (7%)* | *1051* |  |  |  |  |
| Patient 2 | *Pre-ART* | *74 (11%)* | *9,251* | Atriplas | 36 days | No | TB, HSV, KS. Candida intertrigo, Thrush |
|  | Week 4 | *46 (8%)* | *<50* |  |  |  |  |
| Patient 3 | *Pre-ART* | *62 (6%)* | *37,684* | Atripla | 39 days | No | Tb, Thrush |
|  | Week 4 | *55 (7%)* | *<40* |  |  |  |  |
| Patient 4 | *Pre-ART* | *12 (1%)* | *85,831* | Atripla | 105 days | No | TB, HSV, Strongyloides |
|  | *Week 2* | *36 (2%)* | *229* |  |  |  |  |
| Patient 5 | *Pre-ART* | *87 (6%)* | *57,258* | Atripla | 26 days | No | TB, HSV |
|  | *Week 4* | *128 (10%)* | *226* |  |  |  |  |
| Patient 6 | *Pre-ART* | *17(4%)* | *54,209* | Atripla | 61 days | No | TB, Hepatitis B, HSV |
|  | *Week 4* | *32(8%)* | *190* |  |  |  |  |
| Patient 7 | *Pre-ART* | *31 (3%)* | *409,990* | Atripla | 96 days | No | TB, Hepatitis B |
|  | Week 4 | 145 (13%) | 152 |  |  |  |  |
| Patient 8 | Week 4 | 27 (3%) | 280 | Atripla | 35 days | No | TB, Thrush, Schistosomiasis |
| Patient 9 | *Pre-ART* | *20 (3%)* | *233,380* | Atripla | 32 days | No | TB, Schistosomiasis |
|  | Week 8 | 65 (5%) | <40 |  |  |  |  |
