# Supplementary material for: Classical complement and inflammasome activation converge in CD14highCD16- monocytes in HIV associated TB-immune reconstitution inflammatory syndrome
Source: PLoS Pathog. 2021 Mar 31;17(3):e1009435. doi: 10.1371/journal.ppat.1009435 (PMC8041190; doi:10.1371/journal.ppat.1009435)
Supplement: S3 Table — (DOC) [file ppat.1009435.s008.doc]

**Supporting Information**

**S3 Table. List of antibodies used in ImageStream and flow cytometry panels.**

| **Panel name** | **Antibody** | **Clone** | **Fluorochrome** | **Supplier** |
| --- | --- | --- | --- | --- |
| **ImageStream panel** | CD14 | M5E2 | BV605 | Biolegend |
|  | CD16 | 3G8 | PE-Cy7 | Biolegend |
|  | HLA-DR | G46-6 | BV421 | BD |
|  | CD2 | RPA-2.10 | PE | BD |
|  | CD3 | HIT3a | PE | Biolegend |
|  | CD19 | SJ25C1 | PE | BD |
|  | CD20 | 2H7 | PE | BD |
|  | CD56 | B159 | PE | BD |
|  | CD66b | G10F5 | PE | BD |
|  | ASC/TMS1 |  | AF647 | Novus Biologicals |
|  | FLICA | ---- | AF488 | Immunochemistry technologies |
| **Complement - Flow cytometry panel** | CD14 | M5E2 | BV605 | Biolegend |
|  | CD16 | 3G8 | PE-Cy7 | Biolegend |
|  | HLA-DR | L243 | APC-Cy7 | BD |
|  | CD2 | RPA-2.10 | eFluor 450 | Life Technology |
|  | CD3 | UCHT1 | eFluor 450 | Life Technology |
|  | CD19 | SJ25C1 | eFluor 450 | Life Technology |
|  | CD20 | 2H7 | eFluor 450 | Life Technology |
|  | CD56 | HCD56 | BV421 | Biolegend |
|  | CD66b | G10F5 | Pacific Blue | Biolegend |
|  | C1q | polyclonal | FITC | Dako |
|  | C3 | 1H8 | PE | BD |
|  | CD59 | p282(H19) | BV711 | BD |
| **MAC* deposition/**  **caspase-1/4/5 activation - Flow cytometry panel** | CD14 | M5E2 | BV605 | Biolegend |
|  | CD16 | 3G8 | PE-Cy7 | Biolegend |
|  | HLA-DR | G46-6 | BV421 | BD |
|  | CD2 | RPA-2.10 | PE | BD |
|  | CD3 | HIT3a | PE | Biolegend |
|  | CD19 | SJ25C1 | PE | BD |
|  | CD20 | 2H7 | PE | BD |
|  | CD56 | B159 | PE | BD |
|  | CD66b | G10F5 | PE | BD |
|  | C9 | aE11 | FITC | Hycult biotech Inc. |
|  | FLICA | ---- | AF660 | Immunochemistry technologies |

***MAC, membrane attack complex**
